# *In vivo* characterization of an Hfq protein encoded by the *Bacillus anthracis* virulence plasmid pXO1

**DOI:** 10.1186/s12866-017-0973-y

**Published:** 2017-03-14

**Authors:** Andrea B. Keefer, Eugenia K. Asare, Andrei P. Pomerantsev, Mahtab Moayeri, Craig Martens, Stephen F. Porcella, Susan Gottesman, Stephen H. Leppla, Catherine E. Vrentas

**Affiliations:** 1 National Institute of Allergy and Infectious Diseases (NIAID), National Institutes of Health (NIH), Bethesda, MD USA; 2 Department of Biology, Frostburg State University, Frostburg, MD USA; 3 Genomics Unit, Research Technologies Section, Rocky Mountain Laboratories, NIAID, NIH, Hamilton, MT USA; 4 National Cancer Institute, NIH, Bethesda, MD USA; 5 National Animal Disease Center, Agricultural Research Service, U.S. Department of Agriculture, Ames, IA USA

**Keywords:** Hfq, *Bacillus anthracis*, anthrax, mRNA, sRNA, gene expression, pXO1

## Abstract

**Background:**

Bacterial Hfq proteins post-transcriptionally regulate gene expression, primarily by mediating the interaction between sRNAs (small RNAs) and their target mRNAs. The role of Hfq-based regulation has been well defined in Gram-negative bacteria, but comparatively less is known about the impact of Hfq proteins in Gram-positive species. The Gram-positive pathogen *Bacillus anthracis* (causative agent of anthrax) is distinct in that it expresses three homologs of Hfq: Hfq1 and Hfq2 from the chromosome, and Hfq3 from the pXO1 virulence plasmid.

**Results:**

In this study, we utilized overexpression as a strategy to examine the impact of Hfq3 on *B. anthracis* physiology. The increase in Hfq3 protein levels led to anomalous cell shape and chain formation, which manifested as a severe growth defect. This phenotype was specific to *B. anthracis*, as Hfq3 expression in *B. subtilis* at similar levels was not toxic. Toxicity was dependent on residues on the distal face of Hfq3 that are involved in mRNA binding in other bacterial species.

**Conclusions:**

Thus, we hypothesize that Hfq3 interacts with RNA(s) involved in essential functions in the *B. anthracis* cell, leading to increased binding upon overexpression that either sequesters or accelerates degradation of RNAs important for growth. These results not only aid in elucidating the role of Hfq proteins in *B. anthracis*, but also contribute to our current understanding of Hfq in Gram-positive bacteria.

**Electronic supplementary material:**

The online version of this article (doi:10.1186/s12866-017-0973-y) contains supplementary material, which is available to authorized users.

## Background

Hfq proteins primarily function as chaperones in the bacterial cell, facilitating the interaction between small RNAs (sRNAs) and their target mRNAs to regulate gene expression [1, 2]. Hfq has also been implicated in the direct modulation of the expression of certain mRNAs [3]. Hfq proteins are members of the Sm and Sm-like RNA-binding family and generally form hexameric rings that bind RNAs on the distal and proximal faces, as well as on a charged rim patch on the outer edge of the ring [4–8].

The role and impact of Hfq proteins has been well characterized in Gram-negative bacteria, with Hfq deletion strains exhibiting severe growth defects, increased susceptibility to a range of environmental stresses, and decreased virulence [9]. In *E. coli*, Hfq overexpression negatively impacts cell physiology by decreasing FtsZ expression, inhibiting proper cell division [10].

Conversely, the role of Hfq and its associated sRNA regulation has not been fully or clearly elucidated in Gram-positive bacteria, despite the expression of numerous sRNAs in these species. For example, deletion of *hfq* from *Staphylococcus aureus* has no major effects on physiology, and sRNA regulation is not impacted [11]. Similarly, while *Bacillus subtilis* Hfq associates with numerous sRNAs *in vivo* [12], Δ*hfq B. subtilis* strains exhibit only subtle effects on their physiology and transcriptome [13, 14]. In contrast, in *Listeria monocytogenes*, Hfq plays a direct role in modulating stress tolerance and virulence [15] and interacts effectively with specific sRNAs [16]. A recent study in *Clostridium difficile* identified multiple phenotypes associated with the knockdown of *hfq* expression, including the first evidence of an *hfq*-dependent growth phenotype in a Gram-positive species [17].

**Fig. 1:**
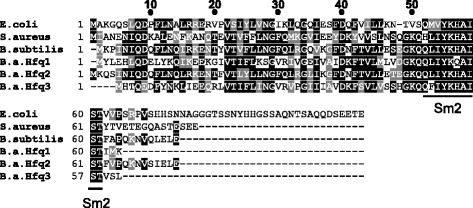
Sequence Alignment of Hfq Proteins. Alignment of *E. coli* Hfq, *S. aureus* Hfq, *B. subtilis* Hfq, and *B. anthracis* Hfq1, Hfq2 and Hfq3 protein sequences using Clustal Omega [43]. BOXSHADE was used to create the image; black shading indicates identical residues, and grey shading denotes biochemically similar residues. The location of the Sm2 RNA binding motif is labeled

Unlike most bacteria, *Bacillus anthracis*, the etiological agent of anthrax, possesses multiple copies of *hfq* within its genome. Two copies are located on the *B. anthracis* chromosome (genes for Hfq1 and Hfq2), and the third (gene for Hfq3) is encoded by the pXO1 virulence plasmid, which also produces the bacterium’s toxin components. Hfq2 is closest in protein sequence to the single copy of Hfq in the close relative *B. subtilis*, whereas Hfq1 and Hfq3 are divergent in sequence from *B. subtilis* Hfq (Fig. 1). Each of the three *hfq* genes is expressed at the mRNA level during the mid-log phase of growth [18].

Our recent work [18] and that of Panda *et al*. [19] expanded the understanding of Hfq structure and function within the *Bacillus* genus by characterization of the structure and function of recombinant *B. anthracis* Hfq proteins. Hfq1 (BA_1656; Hfq2 in [19]) does not form the typical hexameric structure expected for the protein family; instead, it behaves as a monomer that appears to disrupt *E. coli* Hfq’s chaperone activity with a dominant negative phenotype [18]. In contrast, Hfq2 (BA_3842; Hfq1 in [19]) forms the standard hexamer [18]. Purified Hfq3 (GBAA_RS29265) behaves as a mixture of hexamer and monomer, and His-Hfq3 partially complements the *E. coli* Δ*hfq* phenotype [18]. Panda *et al*. [19] reported binding of RNAs to both Hfq2 and Hfq3 (our nomenclature) *in vitro*; however, analysis of Hfq3 binding was limited to a mixed pool of labeled cellular RNAs, and no specific RNA-Hfq3 binding pairs were identified.

In this study, we examine the effects of overexpressing Hfq3 in *Bacillus anthracis*, as a means of identifying potential Hfq3-regulated pathways. We selected Hfq3 for further analysis due to its sequence divergence from other *Bacillus* Hfq proteins, as well as its ability to form hexamers and its presence on one of *B. anthracis*’ virulence plasmids. We observed a severe growth defect associated with the overexpression of Hfq3 in *B. anthracis*, but not in *B. subtilis*. Based on mutational analysis, we determined that this phenotype is dependent on residues on the distal face of Hfq3 that are associated with mRNA binding in other species. Overall, we report a novel phenotype associated with Hfq overexpression in a Gram-positive species, contributing to our current understanding of structure-function relationships of Hfq proteins in Gram-positive bacteria.

## Methods

### Bacterial strains, media, and growth conditions

All bacterial strains, plasmids, and primers used in this study are listed in Additional file 1: Tables S2–S4. TOP10, DH5-α, and SCS110 *E. coli* strains were used for cloning. *B. anthracis* strain Ames 35 (pXO1^+^, pXO2^−^; derived from [20]) and *B. subtilis* strain PY79 [21] were grown in LB broth/plate or NBY broth/plate (containing 0.8% nutrient broth, 0.3% yeast extract, and 0.9% sodium bicarbonate). LB and NBY/0.9% sodium bicarbonate cultures were incubated at 37 °C in air or a CO_2_ environment, respectively. *E. coli* Hfq *in vivo* complementation strains (YN585 derivatives) were grown on MacConkey agar plates (BD Difco; Franklin Lakes, NJ) incubated at 37 °C in air. When appropriate, antibiotics were added to both liquid and solid media: ampicillin (100 μg/mL); kanamycin (15 μg/mL).

### Cloning and transformation

Each *hfq* gene was cloned into the pSW4 shuttle expression vector [22] using the NdeI and BamHI restriction sites. After passage through SCS110 to prepare unmethylated DNA, pSW4 vectors were transformed into *B. anthracis* via electroporation. pSW4 expression vectors were transformed into *B. subtilis* strain PY79 via natural competency using Modified Competency Media (3 mM sodium citrate, 2% glucose, 20 μg/mL ferric ammonium citrate, 0.1% casein hydrolysate, 0.2% potassium glutamate, 100 mM potassium phosphate, 10 mM MgSO_4_) [23].

### Total lysate and western blot analysis

Bacteria were grown in LB or NBY/0.9% sodium bicarbonate cultures in either air (LB) or 15% CO_2_ (NBY). Cells were harvested via centrifugation, and cell pellets were lysed in 1X PBS via beadbeating. Protein concentrations of lysate supernates were estimated via an A_280_ measurement, and gels/blots were normalized by loading equal amounts of total protein per well, which was validated by Coomassie Blue staining. Proteins were separated on 4–20% Tris-glycine gels (Life Technologies; western blot) or 16% Tricine gels (Life Technologies; total lysate). Samples were run as semi-native (not heated) vs. boiled (5 min, 99 °C), and were prepared with either 2X Tris-glycine SDS loading buffer (Life Technologies; final [SDS] 1%) or 2X Tricine SDS loading buffer (Life Technologies; final [SDS] 4%). Both gel systems utilized running buffer containing 0.1% SDS. Following SDS-PAGE, 16% Tricine gels were Coomassie Blue stained, and 4–20% Tris-glycine gels were electroblotted onto nitrocellulose. Membranes were blocked with Odyssey Blocking Buffer (LI-COR Biosciences, Lincoln, NE) and probed with anti-Hfq3 antibodies (1:2000 dilution) overnight at room temperature. An anti-mouse IR800-conjugated secondary antibody (LI-COR Biosciences) was utilized for detection, and blots were visualized on the Odyssey LI-COR infrared scanner.

### Site-directed mutagenesis

Point mutations were introduced into Hfq3 in the pSW4 and pQE80 vectors by the Q5 site-directed mutagenesis kit (New England Biolabs; Ipswich, MA) using the primers listed in Additional file 1: Table S4, as well as the primers in [18] for the Q4A mutation in pQE80. Each mutant gene was fully sequenced in the coding region.

### Microscopy

Samples for transmission electron microscopy (TEM) and differential interference contrast microscopy (DIC) were prepared from LB plate scrapings. For TEM, cells were washed with PBS and resuspended in a 2% paraformaldehyde and 0.1 M sodium cacodylate fixative. Images were collected using a Hitachi H-7500 transmission electron microscope. For DIC imaging, a Leica TCS SP5 confocal microscope DMI 6000 (Leica Microsystems GmbH, Manheim, Germany) was used to collect the images with a 63x oil immersion objective NA 1.4. Images were post-processed using Imaris image processing software (Bitplane, Germany) and Adobe Photoshop 7.0 (Adobe Systems).

### Complementation in *E. coli*

pQE80 His-Hfq3 expression plasmids (Additional file 1: Table S2) were transformed into a Δ*lacZ*Δ*hfq* strain of *E. coli* carrying an *rpoS-lacZ* translational fusion (strain YN585). Resulting strains were streaked to MacConkey (+100 μg/mL ampicillin) agar plates (BD Difco) and incubated for ≈ 16 h at 37 °C to observe color development. Lactose in the MacConkey plates induces constitutive expression of His-Hfqs from the plasmids. β-galactosidase assays were conducted on overnight MacConkey plate scrapings essentially as described in reference [18]. To observe total Hfq protein expression levels, cells were scraped from MacConkey plates and lysed with an 8 M urea buffer (pH 8). Lysates were visualized on 16% Tricine gels by Coomassie Blue staining.

We observed that deletion of the His-tag from the wild-type Hfq3 construct reduced the pink color on MacConkey plates; this may be due to reduction in protein accumulation in the absence of the tag, impacts on other properties of heterologous expression, and/or impacts of the tag on the biochemistry of the protein itself. Therefore, the original His-Hfq3 system for heterologous production in *E. coli* was retained. (We note that all *Bacillus* phenotypes described in this paper are in the absence of any additional tag residues, with the native protein sequence).

### Antibiotic sensitivity and cell autolysis assays

Minimum inhibitory concentration (MIC) testing was performed based on the protocol described in reference [24], with modifications indicated below. LB plate scrapings of individual colonies of Hfq3 or GFP overexpression strains were diluted in LB-Kanamycin (15 μg/ml). Vancomycin and ampicillin were serially diluted in LB-Kanamycin across a 96-well plate with final concentrations ranging from 32–0.06 μg/ml (for vancomycin) and from 32–0.001875 μg/ml (for ampicillin). Hfq3 and GFP overexpression strains were inoculated to a final CFU count of 1.4–6.8 × 10^4^/well. Plates were incubated at 37 °C with shaking at 200 rpm for 19 h, and then visually observed for the presence of turbidity to determine MICs. MICs in Additional file 1: Table S5 represent results for 12 GFP overexpression strain colonies and 21 Hfq3 overexpression strain colonies aggregated across at least three independent experiments.

To monitor cell autolysis, LB plate scrapings of Hfq3 or GFP overexpression strains were resuspended to an OD_600_ of 0.7–1.0 in LB-Kanamycin (15 μg/ml). Due to the small colony size of the Hfq3 strain, replicates for both strains were created from multiple colonies. Cultures were incubated at 37 °C with shaking at 225 rpm for 30 min to resume active growth, followed by addition of Triton X-100 to a final concentration of 0.05% (*v/v*) and continued incubation at 37 °C.

### Animal studies

Female Balb/cJ mice (6–8 weeks old) were purchased from Jackson Laboratories (Bar Harbor, ME). All animal studies were performed in accordance with National Institutes of Health and Animal Welfare Act guidelines on protocol LPD8E, approved by the Animal Care and Use Committee of the National Institute of Allergy and Infectious Diseases, National Institutes of Health.

### Mouse antibodies

To generate mouse polyclonal antibodies against Hfq3, Balb/cJ mice were immunized -12, -9, -6 and -4 weeks prior to terminal serum bleeds. Immunizations were performed subcutaneously with 10–30 μg of purified His-Hfq3. Aluminum hydroxide gel (Alum; Sigma, St. Louis, MO) was used as adjuvant and premixed with antigen at 1:1 (*v/v* Hfq3: Alum). Antibody reactivity and specificity were assessed by western blotting (Additional file 2: Figure S1A, B) and by dilution ELISA. The anti-Hfq3 antibody was specific for purified Hfq3 (as compared to Hfq1 or Hfq2; Additional file 2: Figure S1B). The antibody was able to detect overexpressed Hfq3 in *B. anthracis* cell lysates, but not endogenous levels of Hfq3 in *B. anthracis* lysates (Additional file 2: Figure S2A). We previously demonstrated that Hfq3 is expressed at the RNA level in log-phase *B. anthracis* [18]. Based on western blotting profiles, it is likely that this antibody recognizes a conformational epitope(s), resulting in better recognition of Hfq3 hexamer vs. Hfq3 monomer. The *in vivo* structure of overexpressed Hfq3 as observed by western blot (mix of hexamer and monomer) is consistent with our previous *in vitro* work with purified His-Hfq3 [18].

## Results

### An Hfq3 overexpression system as an investigational tool

Our previous work compared the biochemical properties of the three Hfq proteins of *B. anthracis*. While the Sm2 RNA-binding motif is well conserved in Hfq3, the unstructured C-terminus present in *E. coli* Hfq is significantly truncated (Fig. 1). In searching for physiological roles of these proteins, we did not observe defects in the growth rates of *hfq* deletion strains, including a Δ*hfq1hfq2hfq3* strain [18], consistent with previous findings in *B. subtilis* [14]. This led us to investigate phenotypes linked to overexpression of Hfq proteins in *B. anthracis* as an alternative means of identifying roles of this protein family in Bacilli. Intentional overexpression is a useful genetic tool for the identification of cellular pathways linked to a gene of interest in wild-type cells, and can identify phenotypes that are not evident from deletion analysis in some cases [25].

**Fig. 2:**
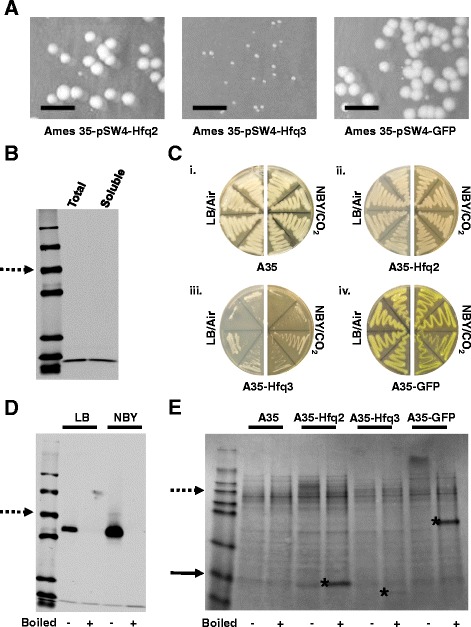
Characterization of Hfq3 Overexpression Toxicity. **a** Anomalous colony morphology of Hfq3 overexpression strain transformants as compared to Hfq2 and GFP overexpression strain transformants (non-inducing/air conditions). Cropped images reflect the same plate area in each case; scale bar 10 mm. **b** Western blot depicting level of Hfq3 whole cell expression vs. soluble expression level (probed with anti-Hfq3 antibody) in an overnight culture grown under inducing conditions. “Total” sample was prepared by lysing cells in 8 M urea, as compared to “soluble” sample, which was prepared by lysing cells by beadbeating in 1X PBS. Equal amounts of total cellular protein were loaded in each well, as verified by a parallel protein gel. Dotted arrow indicates the 50 kDa marker band. **c** Growth of *B. anthracis* strains patched to LB incubated at 37 °C in air or to NBY/bicarbonate incubated at 37 °C in 15% CO_2_, as indicated. (i) Ames 35 parent strain, (ii) Hfq2 overexpression strain, (iii) Hfq3 overexpression strain, and (iv) GFP overexpression strain. **d** Expression of Hfq3 in the absence (LB) and presence (NBY) of inducing conditions. Equal amounts of total soluble protein from cell lysates were subjected to SDS-PAGE and western blotting with anti-Hfq3. Samples were analyzed under semi-native (-) and boiled (+) conditions. Due to the growth phenotype, log-phase OD_600_ values for Hfq3 are difficult to attain, so Hfq3 levels depicted here for both LB and NBY are derived from a 10 mL culture grown to an OD_600_ of 0.14–0.2. Dotted arrow represents the 50 kDa marker band. **e** Total cell lysate gel comparing overexpression levels of Hfq2, Hfq3, and GFP in *B. anthracis* grown under inducing conditions to an OD_600_ of 0.7–1.0; the Hfq3 strain was prepared as described above in panel (**d**). Equal amounts of total soluble protein were subjected to SDS-PAGE. Samples were analyzed under semi-native (-) and boiled (+) conditions. Dotted arrow represents the 55 kDa marker band, solid arrow represents the 7 kDa marker band, and asterisks denote approximate locations of Hfq2, Hfq3, and GFP protein bands

To generate an overexpression system, we prepared a series of pSW4 vectors in which each Hfq gene is under the control of a version of the *B. anthracis* protective antigen promoter. Expression from pSW4 is induced by transition of cells from air/LB media to CO_2_/bicarbonate-containing media (NBY + bicarbonate). Upon transformation of each Hfq overexpression vector into *B. anthracis*, a striking difference was noted in the colony morphology of the Hfq3 overexpression strain as compared to the Hfq2 overexpression strain, as well as to a GFP overexpression strain serving as a control (Fig. 2a). Hfq3 expression-induced toxicity was observed in our earlier work in *E. coli* when His-Hfq3 was expressed from a T7 promoter. In that case, His-Hfq3 was predominantly located in insoluble inclusion bodies [18]. When overexpressed in *B. anthracis* via pSW4, Hfq3 is present in the soluble fraction (Fig. 2b; Additional file 2: Figure S2A), suggesting a toxic effect of soluble Hfq3 in *B. anthracis*.

### Characterization of the Hfq3 toxic growth phenotype in *B. anthracis*

To further characterize the impact of Hfq3 overexpression on *B. anthracis*, we directly compared the growth of Ames 35 (parent strain), Ames 35 overexpressing Hfq2, Ames 35 overexpressing Hfq3, and Ames 35 expressing an unrelated protein (GFP variant). These plates recapitulated the negative impact of Hfq3 overexpression both at 15% CO_2_ (Fig. 2c, iii) and at 5% CO_2_ (Additional file 2: Figure S2B). Overexpressed Hfq3 has a toxic phenotype in air (Fig. 2c, iii, left) that is variable (Fig. 2c, iii, left; Additional file 2: Figure S2E). This can be ascribed to leaky expression from the pSW4 vector, since Hfq3 expression was observed under both non-inducing and inducing conditions, with an increase in Hfq3 production upon induction (compare LB to NBY/CO_2_; Fig. 2d). Under inducing conditions, the overexpressed Hfq3 accumulates to lower levels than overexpressed Hfq2, relative to total cellular protein, as is visible in Fig. 2e. The GFP variant protein is expressed more abundantly than either of the Hfqs (Fig. 2e), indicating that overexpression of proteins in general does not impair growth in *B. anthracis*.

While Hfq2 overexpression did not reproducibly inhibit growth on the plate patches (Fig. 2c; Additional file 2: Figure S2B), about 50% of the Ames 35 pSW4-Hfq2 transformants across 52 individual colonies and 9 experiments exhibited toxicity under inducing conditions (NBY/CO_2_). In parallel experiments, >95% of independent pSW4-Hfq3 transformants exhibited growth defects. This defect is evident after 24 h of growth at 30 °C for the Hfq2 overexpression strain (Additional file 2: Figure S2C). However, we did not observe any evidence on the plates of pSW4-Hfq2 toxicity under non-inducing conditions (low level of Hfq expression, as described in Fig. 2a). Therefore, we propose that overexpression of Hfq2 in *B. anthracis* has a toxic effect once cellular protein levels reach higher concentrations than for Hfq3, which appears to be toxic to the cells even at low, leaky levels of expression. Additionally, we hypothesize that variability in Hfq2 toxicity is due to variations in protein expression levels across independent transformants.

**Fig. 3:**
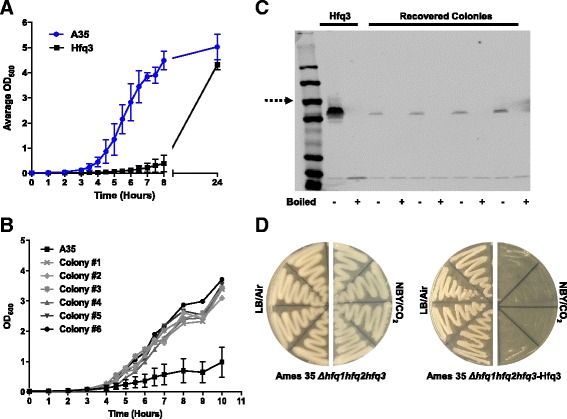
Characterization of Hfq3-associated Toxicity in Liquid Media. Growth curves were performed at 37 °C, 225 rpm, and 15% CO_2_ in NBY/bicarbonate media. **a** Growth comparison of Ames 35 WT (A35; blue) and Ames 35-Hfq3 overexpression strain (Hfq3; black). Data is expressed as the mean of three biological replicates for each strain +/- the standard error of the mean (SEM). **b** Growth comparison of recovered colonies #1–6 to the parent Hfq3 overexpression strain. Error bars reflect standard error of the mean (SEM) of three biological replicates for the parent Hfq3 strain. The doubling time during log phase averaged across the six recovered colonies was 41 ± 1 min. and the doubling time over this time course for the Ames-35 Hfq3 cells was ≈ 90+ min. In contrast, the doubling time for wild-type Ames 35 grown under these conditions (but no antibiotic) in parallel experiments is ≈ 33–35 min. **c** Expression levels of Hfq3 from recovered colonies as compared to the parent Hfq3 overexpression strain. Equal amounts of total soluble protein from cell lysates (as verified by protein gels) were subjected to SDS-PAGE and western blotting with anti-Hfq3. Samples were analyzed under semi-native (-) and boiled (+) conditions. Dotted arrow represents the 50 kDa marker band. Based on ImageJ analysis, Hfq3 expression levels were reduced between 6 and 17-fold as compared to the Hfq3 overexpression parent strain in recovered isolates with detectable protein expression. No protein expression was detectable in isolates carrying the nonsense mutations described in Results. **d** Growth of Ames 35 Δ*hfq1hfq2hfq3* and Ames 35 Δ*hfq1hfq2hfq3* overexpressing Hfq3 patched to LB incubated at 37 °C in air or to NBY/bicarbonate incubated at 37 °C in 15% CO_2_, as indicated. As described in Results, the severity of the phenotype of Hfq3-overexpressing strains in air is variable across independent experiments

A corresponding growth defect in liquid was observed for the Hfq3 overexpression strain (Fig. 3a). Hfq3 overexpression cultures eventually reached near wild-type OD_600_ values after 24 h of growth (Ames 35 WT = 5.0 +/- 0.30; Ames 35-Hfq3 = 4.3 +/- 0.11). Plating of the 24-h cultures on LB-kanamycin vs. LB demonstrated retention of antibiotic resistance, suggesting that recovery of normal growth was not due to plasmid loss. “Recovered” Hfq3-overexpressing colonies (from the plating of 24-h cultures) exhibited a normal growth phenotype from the start of growth (Fig. 3b). Hfq3 expression levels of these colonies were reduced relative to total cellular protein (Fig. 3c; Additional file 2: Figure S2D), suggesting that the recovered phenotype is a consequence of reduced Hfq3 expression levels due to a suppressor mutation(s) within *hfq3* or other regions of the plasmid. Plasmids were prepared from eight “recovered” colonies, and the Hfq3 inserts were sequenced. Seven of the eight clones sequenced successfully; two of these carried a nonsense mutation in residue Q4 of Hfq3, and the other five did not carry any mutations in the Hfq3 reading frame or in the upstream promoter region, nor did the plasmid visibly differ in size on an agarose gel (data not shown). These clones may carry mutations in other regions of the plasmid that result in copy number changes, for example. Due to the rapid accumulation of suppressor mutations in liquid cultures, we have focused on plate-based growth assays in this paper. We note, however, that we cannot rule out the possibility that Hfq3 overexpressing transformants already have accumulated some level of suppressor mutations upon their initial appearance on the plate.

### Distal face residues are required for Hfq3 toxicity

One potential etiology of the Hfq3 overexpression phenotype could be the interaction of overexpressed Hfq3 with endogenous Hfq proteins. However, an Ames 35 Δ*hfq1hfq2hfq3*-Hfq3 overexpression strain retains the toxic phenotype (Fig. 3d; Additional file 2: Figure S2E).

Alternatively, the observed toxicity could be due to increased Hfq3 binding to specific RNAs. To investigate this possibility, we created a set of Hfq3 mutants (in the pSW4 overexpression plasmid) designed to disrupt residues predicted to be necessary for different Hfq-RNA binding faces based on mutagenesis studies from other bacterial species (Table 1). Note that figures are labeled with *B. anthracis* Hfq3 residue numbers; however, the analogous *E. coli* and *S. aureus* residues are listed in Table 1 for comparison with the Hfq mutant literature. Hfq has been demonstrated to bind RNAs via its proximal and distal faces, as well as its rim region [4–7]. In *E. coli*, mutation of residues Q8 or K56 on the proximal face resulted in a severe reduction of Hfq activity, due to disruption of sRNA binding [26, 27]. Effects of mutation of proximal face residue D9 on *E. coli* Hfq function were mixed [26, 28–30], and the D9 residue is linked to a role in the selectivity of sRNA binding, thus facilitating proper annealing [30]. Mutation of *E. coli* residues Y25, I30, or K31 on the distal face had variable effects on Hfq activity, ranging from nearly fully functional (K31) to completely nonfunctional (Y25, I30), due to disruption of interactions with mRNA [26]. Finally, an arginine patch located on the rim of Hfq has been implicated in mediation of mRNA:sRNA interactions [5]; when the patch is mutated in *E. coli*, Hfq experiences a partial to complete loss of function, depending on the characteristics of the specific sRNAs and mRNAs being assayed [26, 27].

**Table 1:** *B. anthracis* Hfq3 mutant nomenclature with analogous Hfq residues in *E. coli and S. aureus*

	*B. anthracis* Hfq3 Mutant	Analogous *E. coli* Hfq Residue	Analogous *S. aureus* Hfq Residue
Proximal Face	Q4A	Q8	Q8
	E5A, D6A (“ED Patch”)	D9, P10	D9, K10
	K53A	K56	K57
Distal Face	R27A	K31	Q31
	F21D	Y25	F25
	V26D	I30	F30
Rim	I12R, E13R (“Rim Rev”)	R16, R17	K16, A17

**Fig. 4:**
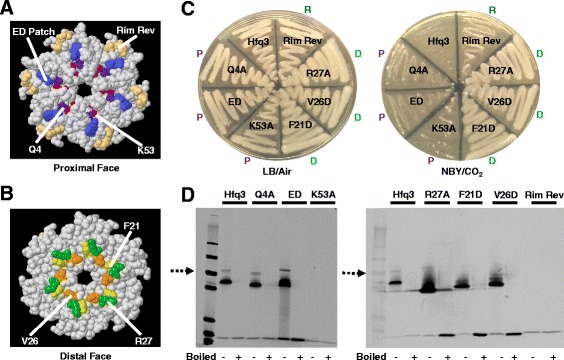
Mutational Analysis of Hfq3. **a**, **b** Jmol-generated images of the proximal and distal faces of *E. coli* Hfq (PDB 3QHS) with mutated residues highlighted and numbered using *B. anthracis* Hfq3 nomenclature. **c** Growth comparison of each Hfq3 mutant patched to LB incubated at 37 °C in air (left) or to NBY/bicarbonate incubated at 37 °C in 15% CO_2_ (right). Mutations are noted as “P” for proximal, “D” for distal, or “R” for rim along the edges of the plate. **d** Expression of each Hfq3 mutant grown under inducing conditions to an OD_600_ of 0.7–1.0. Note that the Hfq3 control was prepared as described in Fig. 2d. Equal amounts of total soluble protein from cell lysates were subjected to SDS-PAGE and western blotting with anti-Hfq3. Samples were analyzed under semi-native (-) and boiled (+) conditions. Dotted arrows represent the 50 kDa marker band

The locations of mutants created by site-directed mutagenesis in *B. anthracis* Hfq3 (Table 1) are depicted on the proximal (Fig. 4a) and distal (Fig. 4b) faces of the *E. coli* Hfq structure. Proximal face residues Q4, E5, D6, and K53, as well as distal face residue R27, were mutated to alanine, and distal face residues F21 and V26 were mutated to aspartic acid (based on previous *E. coli* mutagenesis). We refer to the E5A/D6A mutant as the “ED Patch” mutant [30]. In Hfq3, the rim arginine patch is not present; the *E. coli* patch ‘RRER’ is replaced by ‘IEEQ’. Thus, we created a “rim revertant” construct in which the Hfq3 ‘IE’ sequence was mutated to the *E. coli* Hfq ‘RR’ identity.

The impact of each mutation on the growth of Hfq3-overexpressing Ames 35 cells was examined under inducing conditions (NBY/CO_2_). Mutation of residues on the distal face (F21D, V26D, R27A) resulted in rescue of normal growth, as observed by the plate method (Fig. 4c, right plate; Additional file 2: Figure S3A). In contrast, mutation of key residues on the proximal face of Hfq3 (Q4A, K53A, ED Patch) did not restore normal growth (Fig. 4c; Additional file 2: Figure S3A). Reversion of the rim residues to the *E. coli* sequence would have been expected to increase toxicity, if this site increased binding of critical RNAs; instead, toxicity was somewhat decreased (Fig. 4c; Additional file 2: Figure S3A), which may be due to the disruption in hexamer formation (Fig. 4d). The relative importance of the distal face mutants is suggestive of mRNA binding by Hfq3, as opposed to an mRNA-sRNA pair leading to toxicity.

Western blot analysis confirmed that each of the three Hfq3 distal face mutants is abundantly expressed in *B. anthracis*, indicating that their normal growth is not due to loss of protein expression (Fig. 4d). The K53A (proximal) and rim revertant mutants are expressed, but do not exhibit stable hexamer formation under these gel conditions (Fig. 4d). Similarly, the corresponding K56A *E. coli* Hfq mutant is able to bind RNA, despite apparent lack of hexamer formation as assessed on a semi-native gel [26, 31]. We hypothesize that these mutants form transient hexamers that are capable of RNA binding, but dissociate too quickly under semi-native gel conditions to be observed here. Additionally, the toxicity of the K53A overexpression strain provides evidence that the toxicity of Hfq3 is not due to Hfq aggregation.

### Distal face Hfq3 mutants also disrupt an *E. coli* complementation phenotype

**Fig. 5:**
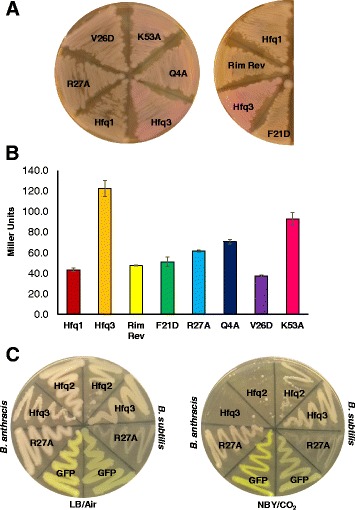
Hfqs Expressed in Other Bacterial Species. **a** Appearance of His-Hfq3 mutants, constitutively expressed from the pQE80 vector in *E. coli* strain YN585, on MacConkey-Ampicillin plates incubated for ≈ 16 h at 37 °C. His-Hfq1 and His-Hfq3-expressing cells are provided for reference. Numbers utilized here and in the text are the *B. anthracis* residue numbers. **b** β-galactosidase assay on MacConkey plate scrapings of YN585 strains expressing His-Hfq3 mutants ≈ 16 h at 37 °C. Bars reflect the average of measurements on three independent cultures, each obtained from an independent transformant; error bars depict +/- standard error of the mean (SEM). β-galactosidase values for reference *E. coli* strains cultured on MacConkey agar (Δ*hfq rpoS*-*lacZ* MG1655; *hfq*+ *rpoS*-*lacZ* MG1655) were 54.4 ± 0.7 and 205 ± 11 Miller Units, respectively. **c** Comparison of growth phenotypes of Hfq2, Hfq3, Hfq3-R27A, and GFP overexpression in *B. anthracis* and *B. subtilis*; plates depict LB incubation at 37 °C in air (*left*) and NBY/bicarbonate incubation at 37 °C in 15% CO_2_ (*right*)

In *E. coli* cells, Hfq is required for translation of the *rpoS* gene transcript; Hfq binds and brings together the 5′ UTR of *rpoS* mRNA with three different sRNAs, freeing the ribosome binding site [32–34]. Previously, we demonstrated that expression of His-Hfq3 in a strain of Δ*hfq*Δ*lac*Z MG1655 *E. coli* cells—in which expression of β-galactosidase is driven from an *rpo*S-*lac*Z translational fusion construct—partially rescued the Δ*hfq* phenotype, as observed by the pink color on MacConkey-lactose agar (Fig. 5a) and in quantitative β-galactosidase assays [18]. His-Hfq1 exhibits no complementation, and serves as a negative control (Fig. 5a) [18]. This is an artificial phenotype, since it (a) examines the impact of a *B. anthracis* Hfq on an *E. coli* RNA regulatory system and (b) utilizes heterologously expressed, His-tagged constructs. However, it provides an opportunity to examine the effects of our set of Hfq3 mutants on an alternative, well-studied phenotype that is linked to RNA binding.

Mutants in His-Hfq3 exhibited defects in complementation of the *E. coli* Δ*hfq* phenotype (Fig. 5a, b), with the largest decrease in complementation observed for the distal face mutants F21D and V26D (Additional file 1: Table S1). The proximal face mutants, as well as the distal face mutant R27A, exhibited a partial loss of the complementation phenotype (Fig. 5b; Additional file 1: Table S1). (However, we note that the results in Fig. 4d suggest that the K53A and rim revertant mutants exhibit unstable hexamer formation, so we have not focused on those mutants here). Denaturing lysate gels reveal that Hfq3 protein accumulates in each of the mutants (Additional file 2: Figure S3B), suggesting that color differences cannot simply be ascribed to lack of His-Hfq3 expression. Therefore, residues involved in the toxic Hfq3 overexpression phenotype were also involved in the complementation phenotype.

### Hfq3 expression in *B. subtilis* does not negatively impact growth

To further probe the physiological mechanism of Hfq3 toxicity, we transformed pSW4-Hfq3 into *B. subtilis* strain PY79. Expression from pSW4 is not induced in *B. subtilis* by CO_2_, but occurs in both air and CO_2_ conditions (observable from the color of GFP expression in Additional file 2: Figure S4A, iv). In contrast to *B. anthracis*, the *B. subtilis* Hfq3 expression strain grew normally (Fig. 5c; Additional file 2: Figure S4A; Additional file 2: Figure S5A). Hfq3 protein was expressed in *B. subtilis* (Additional file 2: Figure S5B, C), indicating that the lack of phenotype is not due to lack of protein expression. The Hfq3-R27A mutant had a change in appearance (opaque to more translucent on streaks); however, the Hfq3 overexpression phenotypes in *B. subtilis* were clearly distinct from the phenotypes in *B. anthracis*. Similar to Hfq2 overexpression in *B. anthracis*, Hfq2 expression in *B. subtilis* yielded an intermediate toxic effect, at a frequency of approximately 28% in 36 transformants (Fig. 5c; Additional file 2: Figure S4A-C). Therefore, the Hfq3 overexpression phenotype exhibits species specificity.

### Overexpression of Hfq3 leads to aberrant *B. anthracis* cellular morphology

**Fig. 6:**
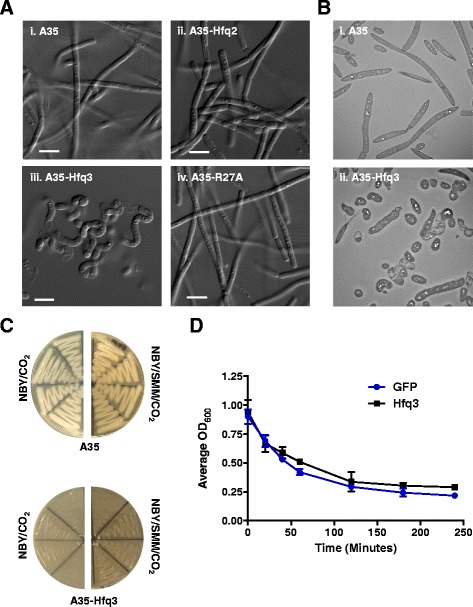
Hfq3 Strain Physiology. Samples for microscopy were prepared from LB plate scrapings for each strain. **a** Differential interference contrast (DIC) microscopy using 63x objective; scale bar 5 μm. (i) Ames 35 parent strain, (ii) Ames 35 overexpressing Hfq2, (iii) Ames 35 overexpressing Hfq3, and (iv) Ames 35 overexpressing Hfq3 distal face mutant R27A. **b** Transmission electron microscopy (TEM) of *B. anthracis* strains at 1500X. (i) Ames 35 parent strain and (ii) Ames 35 overexpressing Hfq3. **c** Growth comparison of Ames 35 parent strain (top) and Hfq3 overexpression strain (*bottom*) patched to NBY/bicarbonate incubated at 37 °C in 15% CO_2_ (*left*) and to NBY/bicarbonate supplemented with 0.5 M sucrose, 16.85 mM maleic acid, and 20 mM MgCl_2_ incubated at 37 °C in 15% CO_2_ (right). **d** Triton X-100 (0.05%)-induced cell autolysis of pSW4-GFP (*blue circles*) and pSW4-Hfq3 (*black squares*) overexpression strains. Error bars reflect standard error of the mean (SEM) of three biological replicates for each strain. Similar results were observed in two additional independent experiments

Based on previous findings that overexpression of *E. coli* Hfq inhibits cell division by decreasing FtsZ expression levels [10], we examined the impact of Hfq3 overexpression on *B. anthracis* cell morphology. Plate scrapings of transformants on LB were analyzed by differential interference contrast microscopy (DIC). The Ames 35-Hfq3 overexpression strain displayed an anomalous chain morphology compared to the Ames 35 parent strain and the Hfq2 overexpression strain, with shortened bacterial chains and an absence of characteristic rod formation (Fig. 6a, i-iii). We emphasize the clear difference between the microscopic appearances of Hfq2 and Hfq3 overexpression strains under non-inducing conditions. In contrast, normal *B. anthracis* chain formation was observed for the distal face mutants (Fig. 6a, iv; Additional file 2: Figure S6). The aberrant shape of the Hfq3-overexpressing cells (Fig. 6b, ii) as compared to Ames 35 (Fig. 6b, i) was also apparent by transmission electron microscopy.

The rounded appearance of the Hfq3-overexpressing cells and the visible defects in cell wall appearance suggest that the increased levels of Hfq3 may impact the activity of MreB and/or the activity of a murein hydrolase in *B. anthracis*. An *mreB* mutant in *B. subtilis* exhibits issues with the maintenance of cell width as well as cell viability; these growth defects were reversible by propagation in media containing magnesium and sucrose [35]. However, supplementation of the NBY/bicarbonate medium by sucrose and magnesium (SMM) did not restore normal growth of the Hfq3 overexpression strain (Fig. 6c).

Finally, to characterize the impact of Hfq3 overexpression on the cell envelope, we compared the physiology of the *B. anthracis* Hfq3 overexpression strain to the *B. anthracis* GFP overexpression strain. Both strains exhibited similar sensitivities (MICs) to both vancomycin and ampicillin (Additional file 1: Table S5). Additionally, both strains exhibited similar sensitivities to lysis by 0.05% Triton X-100 (Fig. 6d). The Hfq3 overexpression strain was capable of spore formation, and the Hfq3 overexpression plasmid was still toxic when transformed into a Spo^−^ *B. anthracis* strain.

## Discussion & conclusions

Previously, our work elucidated the molecular properties of the unusual set of three distinct Hfq proteins produced in the *Bacillus anthracis* cell, including Hfq3, expressed from a gene located on the pXO1 virulence plasmid of the pathogen. Here, we exploited the pSW4 plasmid overexpression system as a tool to examine functions of Hfq3 *in vivo* in *B. anthracis.* A similar overexpression strategy was successfully utilized to identify new binding activities of Hfq in *E. coli* [10, 36], although we note that the *E. coli* system is distinct in its clear and diverse phenotypes observed in the Δ*hfq E. coli* mutant. In *B. anthracis*, we observed a striking toxic effect of Hfq3 overexpression, even at the lower levels observed under LB growth conditions. The toxic effect is dependent on Hfq distal face residues implicated in mRNA binding in *E. coli*, but independent of proximal face residues implicated in small RNA binding. Strikingly, toxicity was species specific and not simply a general effect; overexpression of Hfq3 in *B. subtilis* did not cause the same growth defect.

We propose that increased intracellular concentrations of Hfq3 lead to increased levels of binding of Hfq3-interacting mRNAs in the *B. anthracis* cell. This could interfere with cellular physiology either by sequestering and blocking translation of these mRNAs due to the high Hfq3 concentrations, or, alternatively, by increasing the rate of degradation and cycling of these mRNAs. In *E. coli*, (ARN)_X_ sequences allow for mRNA binding to the distal face of *E. coli* Hfq; binding can then target mRNAs for degradation in some cases [37]. The results for Hfq3 overexpression in *B. subtilis* seem to suggest that the toxic phenotype is not due to general sequestration of all mRNAs. The distal face mutagenesis does not directly demonstrate RNA binding, and it is alternatively possible that the Hfq3 overexpression phenotype is due to protein-protein interactions. However, the importance of residues previously demonstrated to disrupt mRNA binding to *E. coli* Hfq is suggestive of an mRNA binding mechanism in the Hfq3-overexpressing cells.

A recent study in *Clostridium difficile*—a species in which Hfq knockdown leads to significant growth defects (unique for Gram-positive bacteria)—identified changes in transcript levels of genes involved in cell wall structure and transport in the absence of Hfq [17]. Importantly, Hfq depletion in *C. difficile* led to cell elongation and cell wall deformation, leading Boudry *et al*. [17] to propose that Hfq regulation influences *C. difficile* cell wall composition. While the Δ*hfq* strain of *B. anthracis* used here exhibits normal growth in rich media, our findings suggest that Hfqs may be playing more subtle roles in the physiology of *B. anthracis*. The collapse of normal cell shape and chain formation in the Hfq3 overexpressing cells could be consistent with an impact on cell wall formation and cell division, correlating with some of the functions in *C. difficile*. Alternatively, the toxic phenotype could be due to other cellular impacts, such as loss of membrane integrity or sequestration of RNAs involved in other essential cell functions. The results of the antibiotic sensitivity and autolysis assays suggest that the phenotype does not involve differences in peptidoglycan crosslinking/teichoic acid structure [38–41].

While overexpression of Hfq2 appears to lead to toxicity as well, the Hfq3 phenotype is distinct in its presence at lower relative levels of protein expression, as observed from the difference in cellular appearance under air (non-inducing) conditions. The Hfq2 phenotype may be the result of weaker binding of the same RNA(s) to the Hfq2 hexamer when the protein reaches higher levels in the cell. We note that we cannot rule out the possibility that growth of the Hfq2 overexpression strain reflects rapid accumulation and growth of suppressor mutants.

Combined with the findings in *B. anthracis*, our mutational analysis in *E. coli* strengthens the argument for the importance of the distal face in Hfq3’s functional capabilities. Additionally, the difference in the Hfq3 rim sequence is suggestive of differences between Hfq3 and *E. coli* Hfq in their abilities to bring together sRNAs and their target mRNAs for regulation. Previously, we modeled the structure and charges of the Hfq3 hexamer, based on its amino acid sequence [18]. The proximal face is predicted to have an inner ring of positive charges, similar to *S. aureus* Hfq, but also exhibits significant patches of RNA-repelling negative charge, in contrast to the findings from *E. coli* Hfq’s crystal structure. The Hfq3 distal face is predicted to have a greater distribution of positive charge that may be favorable for mRNA binding. Another potential variable is the very short length of the Hfq3 C-terminal tail, which still allows for hexamer formation, but may impact the sRNA-binding function of Hfq3.

Overall, the finding of a *B. anthracis*-specific overexpression phenotype for Hfq3 is novel for a Gram-positive species, and provides an impetus for the further study of Hfq3-associated functions in *B. anthracis*. The next steps for research on the function(s) of this protein will be to elucidate the impact of reduction in Hfq3 levels on the *B. anthracis* transcriptome, with the results presented here as a point of comparison for identification of transcripts potentially linked to impacts on cellular structure or metabolism. Additionally, the results presented here provide the foundation for a much more targeted exploration of potential phenotypes of Δ*hfq3 B. anthracis* cells. Further analysis is important in understanding the level of physiological relevance of the findings from the overexpression system. To this end, we conducted a preliminary transcriptome analysis of an *hfq3* deletion mutant of Ames 35, as compared to the wild-type strain grown in NBY + bicarbonate (in CO_2_) to late log phase (Additional file 3: Experiment 1). These initial results suggest that there are gene expression differences in the absence of Hfq3 that may have impacts on cellular physiology. Of potential future interest would also be the role of Hfqs in the context of the pXO2 plasmid, present in fully virulent strains of *B. anthracis* and responsible for capsule formation.

Finally, we propose that the relative importance of distal face mRNA-dependent mechanisms, as opposed to sRNA-mRNA pairing mechanisms, may be of broader importance in RNA regulation in Gram-positive species. Such mechanisms would be consistent with the small subset of sRNA-independent mechanisms for post-transcriptional regulation of certain mRNAs in *E. coli* [37, 42].

## Additional files

Additional file 1: Table S1. β-galactosidase assay result summary. Results from two independent trials of the β-galactosidase assay depicted in Fig. 5b. (Each trial involved triplicate independent transformants for each mutant). In these assays, Miller units for each sample were corrected by first subtracting the value for the Hfq1 strain (used as a negative control), and then were compared to the corrected value for the wild-type Hfq3 strain, set to 100%. Mutants with negative values exhibited lower expression than the Hfq1 control strain. While we observed some variability in the signal as a percentage of the wild-type Hfq3 signal between trials, the same rank order of mutant expression levels was observed in each trial. **Table S2.** Plasmids created in this study. **Table S3.** Bacterial strains utilized and/or created in this study. **Table S4.** Primers utilized in this study. **Table S5.** MIC (μg/mL) of cell wall-targeting antibiotics for Hfq3 and GFP overexpression strains. (PDF 436 kb)
Additional file 2: Figure S1. Characterization of Anti-Hfq3 Antibodies. (A) Western blot of reactivity of anti-Hfq3 antibody against purified His-Hfq3. (B) Western blot assessing cross-reactivity of anti-Hfq3 antibody with other *B. anthracis* His-Hfqs. **Figure S2.** Further Characterization of *B. anthracis* Hfq2 and Hfq3 Overexpression Strains. (A) Western blot of Hfq3 expression in the Ames 35 parent strain vs. the Hfq3 overexpression strain. (B) Growth comparison of Hfq2 and Hfq3 overexpression strains at 37 °C. (C) Growth comparison of Hfq2 overexpression strain to Ames 35 parent strain at 30 °C; NBY/bicarbonate in 15% CO_2_ for ≈ 24 h. (D) Total lysate gel comparing Hfq3 protein levels of the Hfq3 overexpression strain to the “recovered” strains depicted in Fig. 3b, c. (E) Further characterization of effect of Δ*hfq123* background on the impact of Hfq3 overexpression. **Figure S3.** Additional Analysis of Hfq3 Mutants. (A) Growth phenotype comparison of Ames 35-Hfq3 mutant overexpression strains. (B) Denaturing lysate gel of *E. coli* His-Hfq3 overexpression strains from Fig. 5a. **Figure S4**. Additional Characterization of Effects of Hfq3 Expression in *B. subtilis*. (A) Growth of *B. subtilis* overexpression strains at 37 °C. (B) Growth phenotypes of Hfq2, Hfq3, R27A, and GFP overexpression in *B. anthracis* and *B. subtilis*. (C) Growth of independent transformants of Hfq2 *B. subtilis* overexpression strain; NBY/bicarbonate at 37 °C in 15% CO_2_. **Figure S5.** Further Investigation of Hfq3 Expression in *B. subtilis*. (A) Light microscopy of *B. subtilis* overexpression strains, grown under inducing conditions, at 20X magnification. (B) Western blot of expression levels of Hfq3 when recombinantly overexpressed in *B. anthracis* (A35) vs. *B. subtilis* (PY79). (C) Total lysate gel comparing overexpression levels of Hfq2, Hfq3, and GFP in *B. subtilis* overexpression strains grown under inducing conditions. **Figure S6**. Further Microscopic Characterization of Hfq3 Distal Face Mutants. Differential interference contrast microscopy on cells grown in non-inducing conditions was performed as in Fig. 6a.
Additional file 3: Experiment 1. RNA-Seq Comparison of Wild-type *B. anthracis* to Δ*hfq3 B. anthracis*. (PDF 649 kb)
