# Supplementary material for: In vivo characterization of an Hfq protein encoded by the Bacillus anthracis virulence plasmid pXO1
Source: BMC Microbiol. 2017 Mar 14;17:63. doi: 10.1186/s12866-017-0973-y (PMC5348863; doi:10.1186/s12866-017-0973-y)
Supplement: Additional file 1: Table S1. — β-galactosidase assay result summary. Results from two independent trials of the β-galactosidase assay depicted in Fig. 5b. (Each trial involved triplicate independent transformants for each mutant). In these assays, Miller units for each sample were corrected by first subtracting the value for the Hfq1 strain (used as a negative control), and then were compared to the corrected value for the wild-type Hfq3 strain, set to 100%. Mutants with negative values exhibited lower expression than the Hfq1 control strain. While we observed some variability in the signal as a percentage of the wild-type Hfq3 signal between trials, the same rank order of mutant expression levels was observed in each trial. Table S2. Plasmids created in this study. Table S3. Bacterial strains utilized and/or created in this study. Table S4. Primers utilized in this study. Table S5. MIC (μg/mL) of cell wall-targeting antibiotics for Hfq3 and GFP overexpression strains. (PDF 436 kb) [file 12866_2017_973_MOESM1_ESM.pdf]

Additional file 1

Table S1. β-galactosidase assay result summary. Results from two independent trials of the β-galactosidase assay depicted in Fig. 5B. (Each trial involved triplicate independent transformants for each mutant). In these assays, Miller units for each sample were corrected by first subtracting the value for the Hfq1 strain (used as a negative control), and then were compared to the corrected value for the wild-type Hfq3 strain, set to 100%. Mutants with negative values exhibited lower expression than the Hfq1 control strain. While we observed some variability in the signal as a percentage of the wild-type Hfq3 signal between trials, the same rank order of mutant expression levels was observed in each trial.

Hfq3 Mutant	Percent of β-galactosidase Signal, Trial 1	Percent of β-galactosidase Signal, Trial 2
K53A	107	63
Q4A	68	35
R27A	23	23
F21D	20	10
Rim Revertant	10	5
V26D	-5	-8

Table S2. Plasmids created in this study.

Plasmid Name	Construction	Description	Antibiotic Resistance	Origin
pABK001	pSW4-Hfq3 (cloning between *Nde*I and *Bam*HI)	For sodium bicarbonate and CO_2 driven expression	Kan^R	This work
pABK002	pSW4-Hfq2 (cloning between *Nde*I and *Bam*HI)	For sodium bicarbonate and CO_2 driven expression	Kan^R	This work
pABK003	pSW4-Hfq3-Q4A	For sodium bicarbonate and CO_2 driven expression	Kan^R	NEB Q5 mutagenesis

pABK004	pSW4-Hfq3-K53A	For sodium bicarbonate and CO_2 driven expression	Kan^R	NEB Q5 mutagenesis
pABK005	pSW4-Hfq3-ED Patch	For sodium bicarbonate and CO_2 driven expression	Kan^R	NEB Q5 mutagenesis
pABK006	pSW4-Hfq3-F21D	For sodium bicarbonate and CO_2 driven expression	Kan^R	NEB Q5 mutagenesis
pABK007	pSW4-Hfq3-V26D	For sodium bicarbonate and CO_2 driven expression	Kan^R	NEB Q5 mutagenesis
pABK008	pSW4-Hfq3-R27A	For sodium bicarbonate and CO_2 driven expression	Kan^R	NEB Q5 mutagenesis
pABK009	pSW4-Hfq3-Rim	For sodium bicarbonate and CO_2 driven expression	Kan^R	NEB Q5 mutagenesis
pABK010	pSW4-GFP	For sodium bicarbonate and CO_2 driven expression	Kan^R	DNA2.0 (Newark, CA)
pCV001	pQE80-T5-His-Hfq1 (cloning between *Bam*HI and *Sal*I)	For *in vivo* experiments in *E. coli*	Amp^R	(Vrentas et al., 2015)
pCV003	pQE80-T5-His-Hfq3 (cloning between *Bam*HI and *Sal*I)	For *in vivo* experiments in *E. coli*	Amp^R	(Vrentas et al., 2015)
pCV016	pQE80-His-Hfq3-Q4A	For *in vivo* experiments in *E. coli*	Amp^R	(Vrentas et al., 2015)
pABK011	pQE80-His-Hfq3-K53A	For *in vivo* experiments in *E. coli*	Amp^R	NEB Q5 mutagenesis
pABK012	pQE80-His-Hfq3-F21D	For *in vivo* experiments in *E. coli*	Amp^R	NEB Q5 mutagenesis

pABK013	pQE80-His-Hfq3-V26D	For *in vivo* experiments in *E. coli*	Amp^R	NEB Q5 mutagenesis
pABK014	pQE80-His-Hfq3-R27A	For *in vivo* experiments in *E. coli*	Amp^R	NEB Q5 mutagenesis
pABK015	pQE80-His-Hfq3-Rim	For *in vivo* experiments in *E. coli*	Amp^R	NEB Q5 mutagenesis

Table S3. Bacterial strains utilized and/or created in this study.

Strain Name	Genetic Background	Other Plasmids and Antibiotic Resistance	Source
TOP10	*E. coli*	None	Thermo Fisher
SCS110	*E. coli*	None	Agilent Technologies
Ames 35	*B. anthracis* $pXO1^+$, $pXO2^-$	None	Lab Collection, derived from Green *et al.* (1985)
Ames 35 Δ*hfq*	*B. anthracis* $pXO1^+$, $pXO2^-$ Δ*hfq1hfq2hfq3*	None	Lab Collection, derived from Green *et al.* (1985)
PY79	*B. subtilis*	None	(Zeigler et al., 2008)
ABK001	Ames 35	pSW4-Hfq3; Kan^R	This work
ABK002	Ames 35	pSW4-Hfq2; Kan^R	This work
ABK003	Ames 35	pSW4-Hfq3-Q4A; Kan^R	This work
ABK004	Ames 35	pSW4-Hfq3-K53A; Kan^R	This work
ABK005	Ames 35	pSW4-Hfq3-ED Patch; Kan^R	This work
ABK006	Ames 35	pSW4-Hfq3-F21D; Kan^R	This work
ABK007	Ames 35	pSW4-Hfq3-V26D; Kan^R	This work
ABK008	Ames 35	pSW4-Hfq3-R27A; Kan^R	This work

ABK009	Ames 35	pSW4-Hfq3-Rim; Kan^R	This work
ABK010	Ames 35	pSW4-GFP; Kan^R	This work
YN585	*E. coli* MG1655 *rpoS-lacZ hfq*::*cat*	Cm^R	S. Gottesman Lab (Wassarman et al., 2001)
CEV017	YN585	pQE80-His-Hfq1; Amp^R	(Vrentas et al., 2015)
CEV019	YN585	pQE80-His-Hfq3; Amp^R	(Vrentas et al., 2015)
CEV028	YN585	pQE80-His-Hfq3-Q4A; Amp^R	(Vrentas et al., 2015)
ABK011	YN585	pQE80-His-Hfq3-K53A; Amp^R	This work
ABK012	YN585	pQE80-His-Hfq3-F21D; Amp^R	This work
ABK013	YN585	pQE80-His-Hfq3-V26D; Amp^R	This work
ABK014	YN585	pQE80-His-Hfq3-R27A; Amp^R	This work
ABK015	YN585	pQE80-His-Hfq3-Rim; Amp^R	This work

Table S4. Primers utilized in this study.

**Primer Name**	**Sequence (5'--> 3')**	**Primer Use**
Pr001	AAACGGCGTAGCAGTACCTGGAATC	Forward Primer, Q5 mutagenesis, Hfq3-R27A
Pr002	ATTAAAAATATCGTTACTAATCTTTGTTC	Reverse Primer, Q5 mutagenesis, Hfq3-R27A

Pr003	GATGCATACAGCAGAAGATTTTTACAACAAATTAATAG	Forward Primer, Q5 mutagenesis, pSW4-Hfq3-Q4A
Pr004	ATATGCGTTCTCCTTTTTG	Reverse Primer, Q5 mutagenesis, pSW4-Hfq3-Q4A
CEV019	ATGCATACAGCAGAAGATTTTTACAAC	Forward Primer, Q5 mutagenesis, pQE80-Hfq3-Q4A
CEV020	GGATCCGTGATGGTGATGGTGATG	Reverse Primer, Q5 mutagenesis, pQE80-Hfq3-Q4A
Pr005	CAACAAATTACGTCGTGAACAAAGATTAGTAACGATATTTTTAATAAACGGC	Forward Primer, Q5 mutagenesis, Hfq3-Rim
Pr006	TAAAAATCTTCTTGTGTATGCATTTATAAAG	Reverse Primer, Q5 mutagenesis, Hfq3-Rim
Pr007	ATTCATTTATGCGCATGCTATTTCAACAG	Forward Primer, Q5 mutagenesis, Hfq3-K53A
Pr008	TGTTGCTTTCCATGACTG	Reverse Primer, Q5 mutagenesis, Hfq3-K53A
Pr009	AGTAACGATAGATTTAATAAACGGCGTAAG	Forward Primer, Q5 mutagenesis, Hfq3-F21D

Pr010	AATCTTTGTTCCTCTATTAATTTG	Reverse Primer, Q5 mutagenesis, Hfq3-F21D
Pr011	AATAAACGGCGATAGAGTACCTG	Forward Primer, Q5 mutagenesis, Hfq3-V26D
Pr012	AAAAATATCGTTACTAATCTTTGTTC	Reverse Primer, Q5 mutagenesis, Hfq3-V26D
Pr013	GCATACACAAGCAGCATTTTACAACAAATTAATAG	Forward Primer, Q5 mutagenesis, Hfq3-ED Patch
Pr014	ATCATATGCGTTCTCCTTTTTG	Reverse Primer, Q5 mutagenesis, Hfq3-ED Patch

Table S5. MIC (µg/mL) of cell wall-targeting antibiotics for Hfq3 and GFP overexpression strains.

Antibiotic	Strain	Plasmid	MIC (µg/ml)
Ampicillin	Ames 35	pSW4-Hfq3	≤0.06-0.125
	Ames 35	pSW4-GFP	0.03-0.125
Vancomycin	Ames 35	pSW4-Hfq3	1.0-2.0
	Ames 35	pSW4-GFP	2.0-4.0

Supplemental References

Green BD, Battisti L, Koehler TM, Thorne CB, Ivins BE. Demonstration of a capsule plasmid in *Bacillus anthracis*. Infect Immun*.*1985*;*49:291-7.

Vrentas C, Ghirlando R, Keefer A, Hu Z, Tomczak A, Gittis A, Garboczi DN, Gottesman S, Leppla SH. Hfqs in *Bacillus anthracis*: Role of protein sequence variation in the structure and function of proteins in the Hfq family. Protein Sci. 2015*;*24:1808-19.

Wassarman KM, Repoila F, Rosenow C, Storz G, Gottesman S. Identification of novel small RNAs using comparative genomics and microarrays. Genes Dev. 2001*;*15:1637-51.

Zeigler DR, Prágai Z, Rodriguez S, Chevreux B, Muffler A, Albert T, Bai R, Wyss M, Perkins JB. The origins of 168, W23, and other *Bacillus subtilis* legacy strains. J Bacteriol. 2008;190:6983-95.
