# Supplementary figures and images for: In vivo characterization of an Hfq protein encoded by the Bacillus anthracis virulence plasmid pXO1
Source: BMC Microbiol. 2017 Mar 14;17:63. doi: 10.1186/s12866-017-0973-y (PMC5348863; doi:10.1186/s12866-017-0973-y)

A.

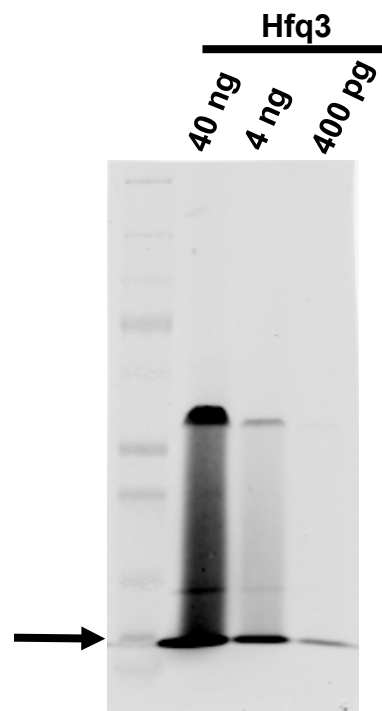

B.

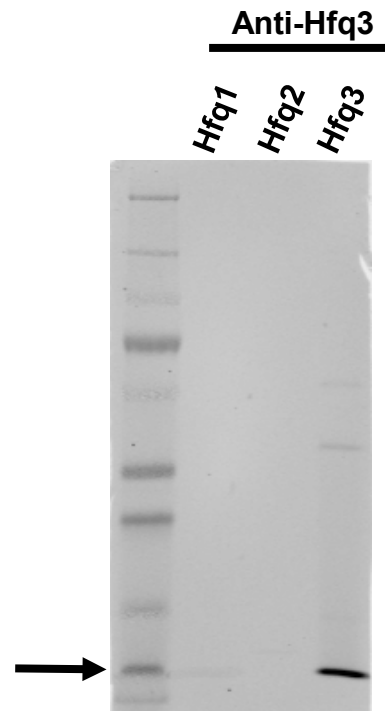

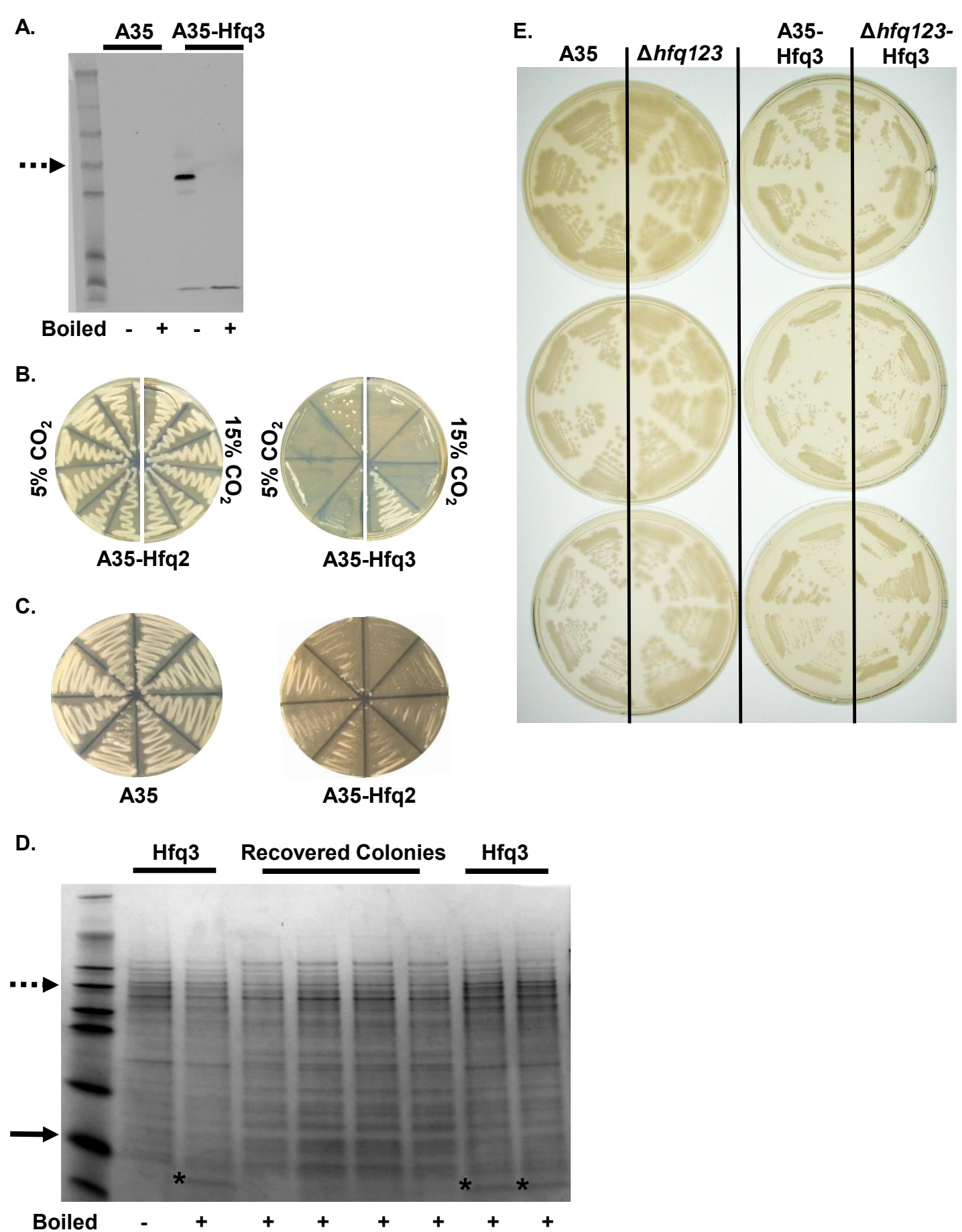

Figure S2

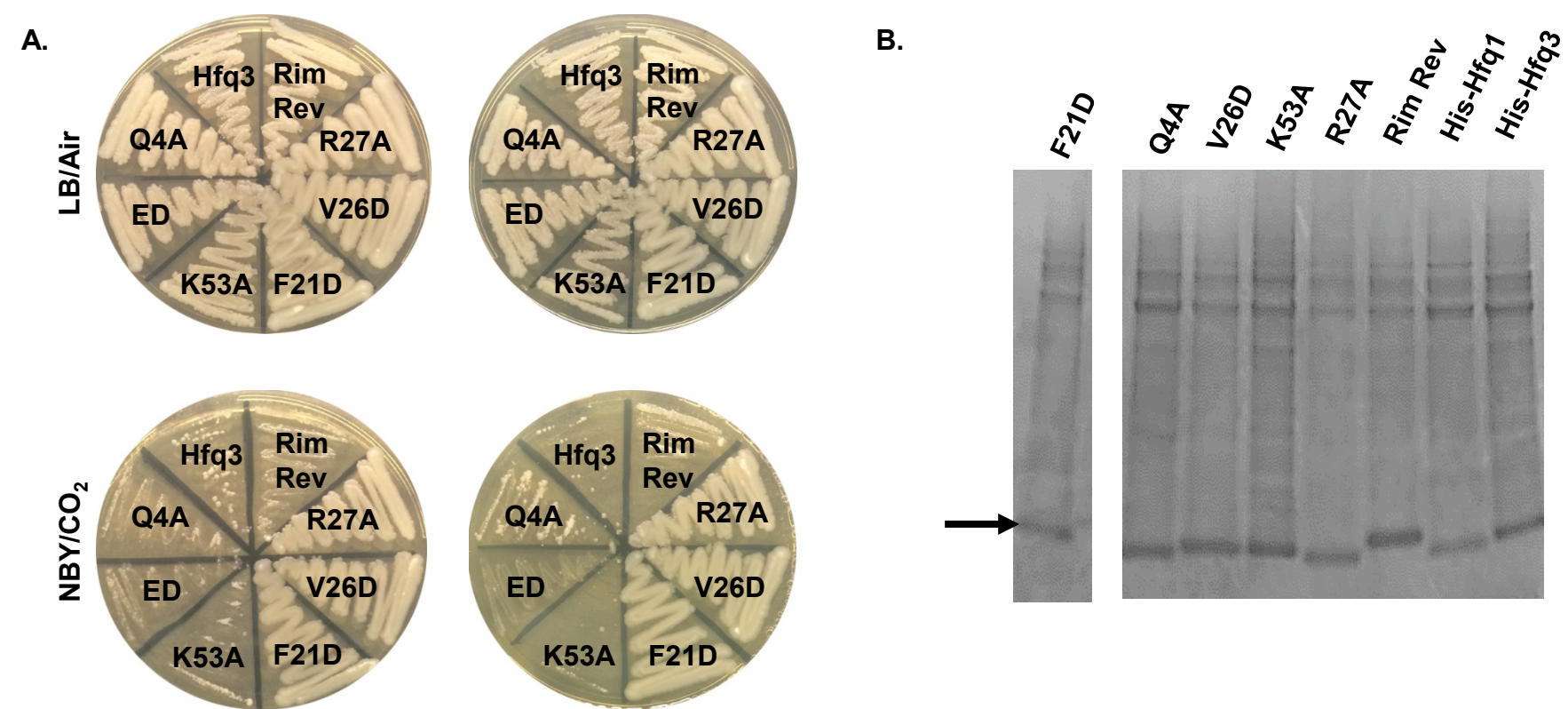

Figure S3

A.

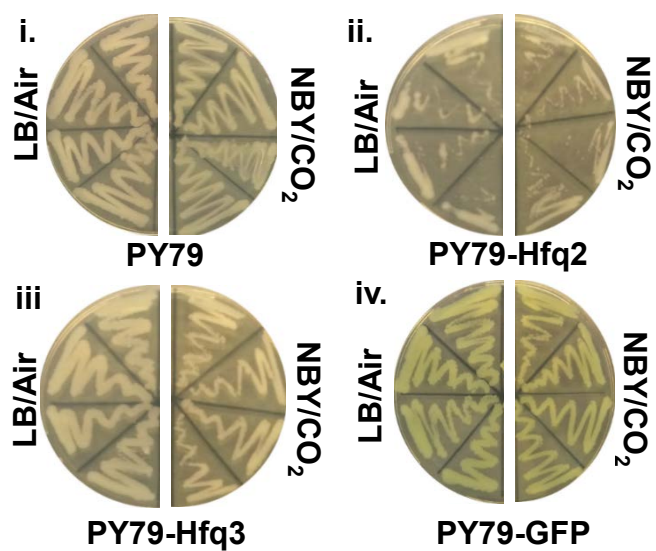

B.

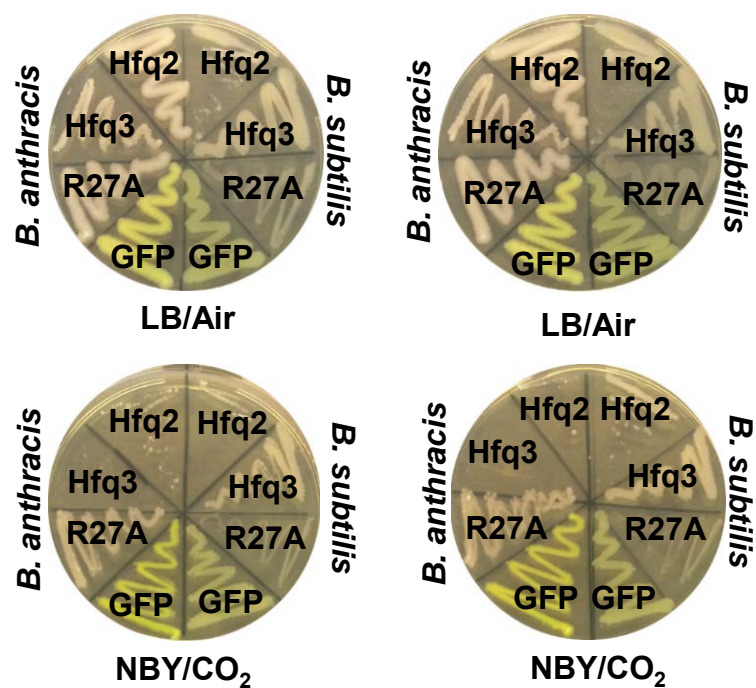

C.

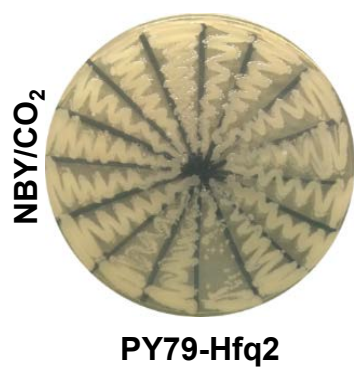

Figure S4

A.

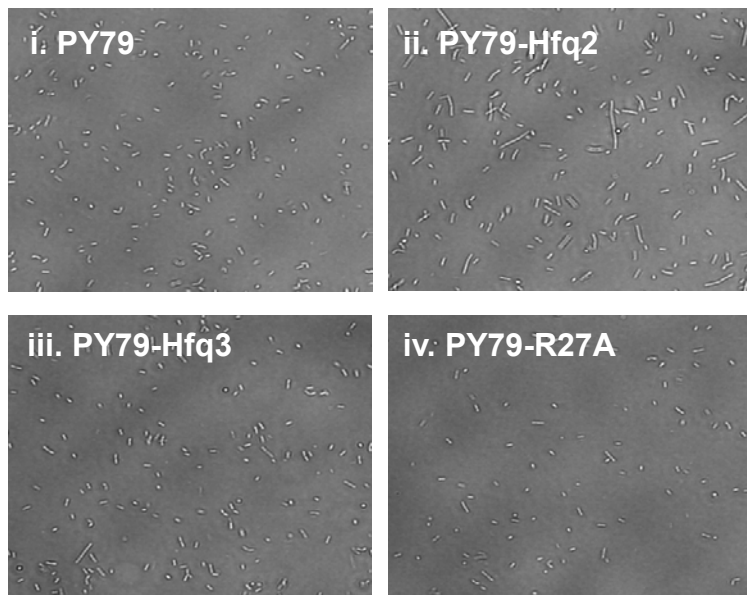

B.

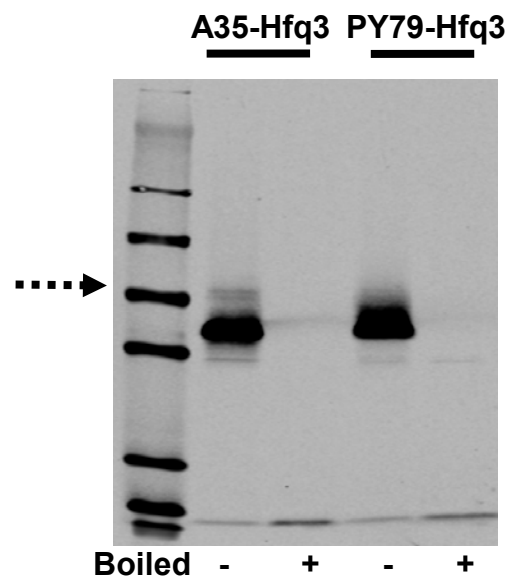

C.

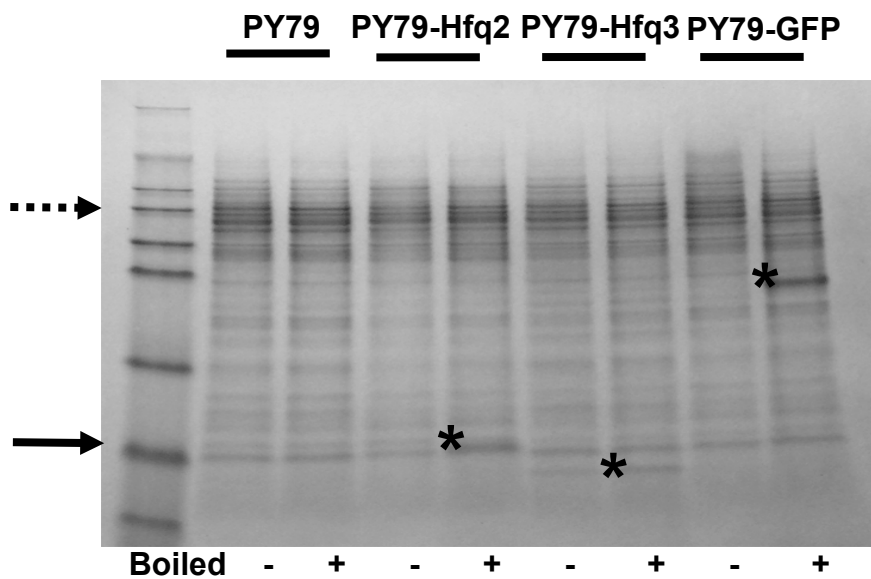

Figure S5

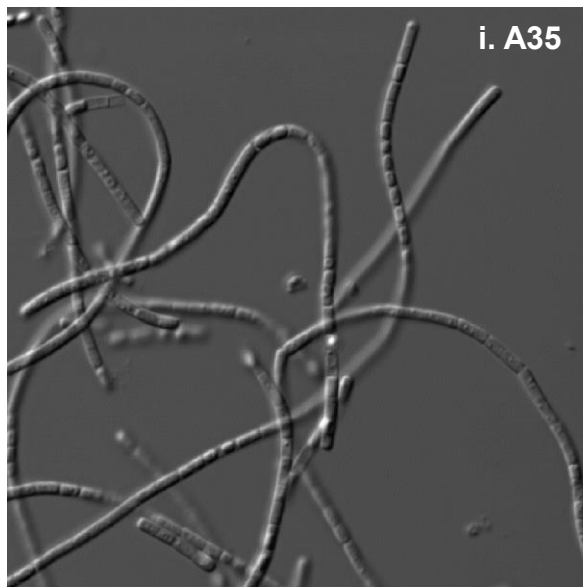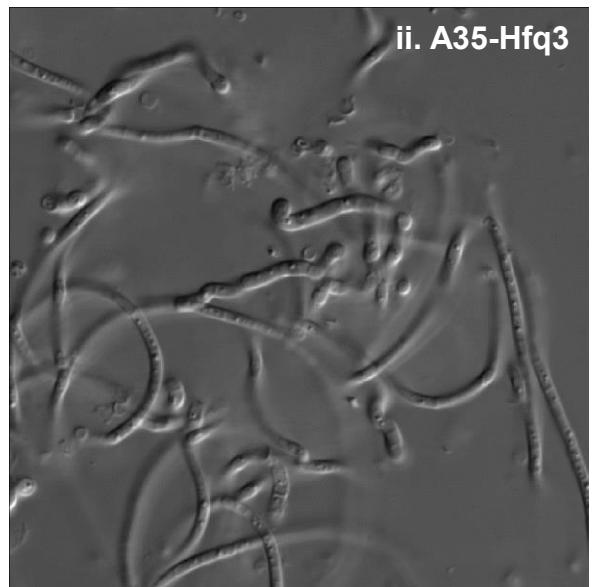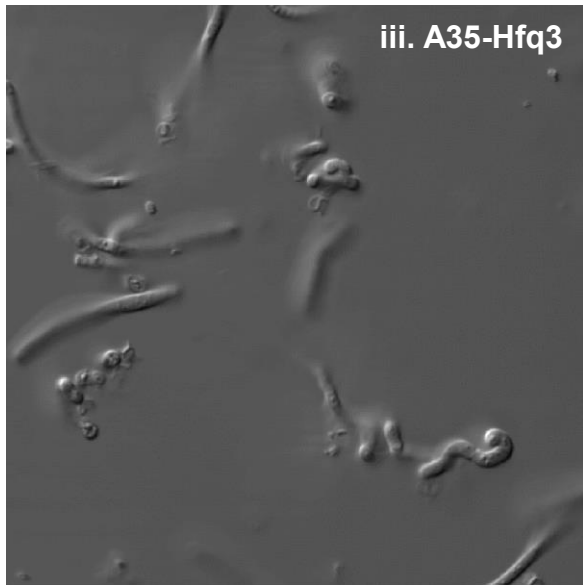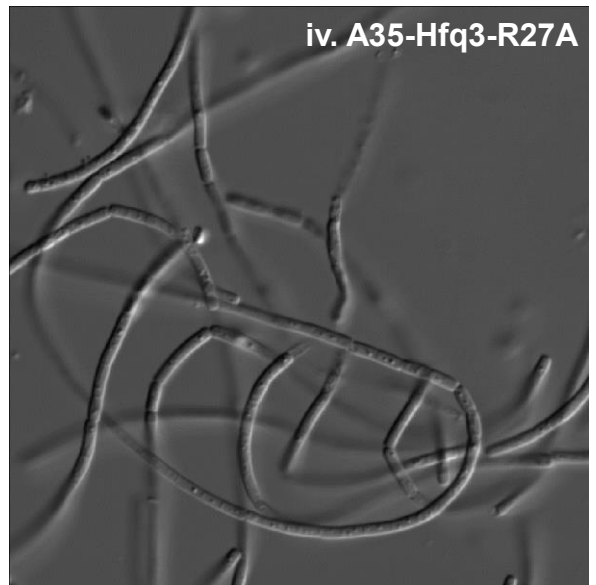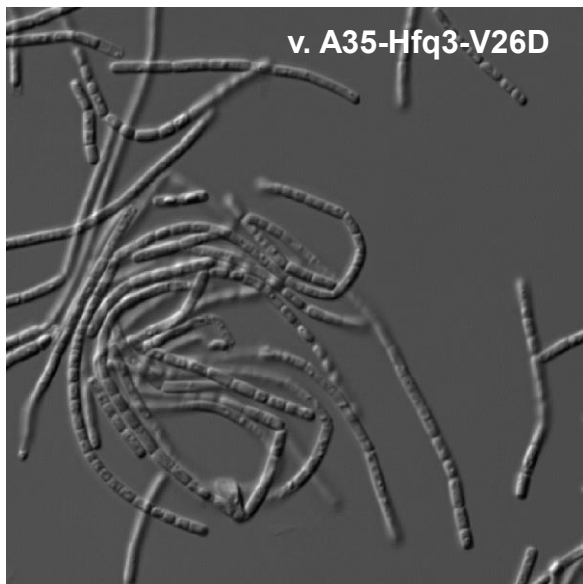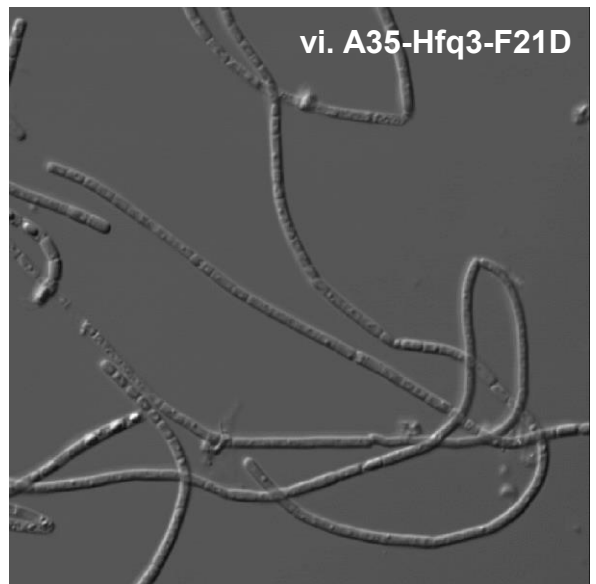

Figure S6

Supplement: Additional file 2: Figure S1. — Characterization of Anti-Hfq3 Antibodies. (A) Western blot of reactivity of anti-Hfq3 antibody against purified His-Hfq3. (B) Western blot assessing cross-reactivity of anti-Hfq3 antibody with other B. anthracis His-Hfqs. Figure S2. Further Characterization of B. anthracis Hfq2 and Hfq3 Overexpression Strains. (A) Western blot of Hfq3 expression in the Ames 35 parent strain vs. the Hfq3 overexpression strain. (B) Growth comparison of Hfq2 and Hfq3 overexpression strains at 37 °C. (C) Growth comparison of Hfq2 overexpression strain to Ames 35 parent strain at 30 °C; NBY/bicarbonate in 15% CO2 for ≈ 24 h. (D) Total lysate gel comparing Hfq3 protein levels of the Hfq3 overexpression strain to the “recovered” strains depicted in Fig. 3b, c. (E) Further characterization of effect of Δhfq123 background on the impact of Hfq3 overexpression. Figure S3. Additional Analysis of Hfq3 Mutants. (A) Growth phenotype comparison of Ames 35-Hfq3 mutant overexpression strains. (B) Denaturing lysate gel of E. coli His-Hfq3 overexpression strains from Fig. 5a. Figure S4. Additional Characterization of Effects of Hfq3 Expression in B. subtilis. (A) Growth of B. subtilis overexpression strains at 37 °C. (B) Growth phenotypes of Hfq2, Hfq3, R27A, and GFP overexpression in B. anthracis and B. subtilis. (C) Growth of independent transformants of Hfq2 B. subtilis overexpression strain; NBY/bicarbonate at 37 °C in 15% CO2. Figure S5. Further Investigation of Hfq3 Expression in B. subtilis. (A) Light microscopy of B. subtilis overexpression strains, grown under inducing conditions, at 20X magnification. (B) Western blot of expression levels of Hfq3 when recombinantly overexpressed in B. anthracis (A35) vs. B. subtilis (PY79). (C) Total lysate gel comparing overexpression levels of Hfq2, Hfq3, and GFP in B. subtilis overexpression strains grown under inducing conditions. Figure S6. Further Microscopic Characterization of Hfq3 Distal Face Mutants. Differential interference contrast microscopy on [file 12866_2017_973_MOESM2_ESM.pdf]
