# Supplementary material for: In vivo characterization of an Hfq protein encoded by the Bacillus anthracis virulence plasmid pXO1
Source: BMC Microbiol. 2017 Mar 14;17:63. doi: 10.1186/s12866-017-0973-y (PMC5348863; doi:10.1186/s12866-017-0973-y)
Supplement: Additional file 3: — Experiment 1. RNA-Seq Comparison of Wild-type B. anthracis to Δhfq3 B. anthracis. (PDF 649 kb) [file 12866_2017_973_MOESM3_ESM.pdf]

Additional file 3: Experiment 1. RNA-Seq Comparison of Wild-type *B. anthracis* to Δ*hfq3 B. anthracis*

Strain Information: The wild-type (parent) strain used in these experiments was the same Ames 35 strain described in the main paper. A deletion of the *hfq3* gene on the pXO1 plasmid was created by *loxP* recombination using a spectinomycin resistance cassette flanked by PCR fragments from the *hfq3* gene; the associated Δ*hfq1hfq2hfq3* mutant derived from this deletion was previously reported in [1]. Three independent colonies from the Ames 35 glycerol stock and the Ames 35-ΔHfq3 glycerol stock were selected from an LB streak plate, cultured overnight in air in LB broth, and inoculated to an OD_{600} < 0.02 in 10 ml NBY + bicarbonate (as described in the main Methods). Cultures were grown to OD_{600} values of 0.9-1.2 in a 15% CO_2 environment, mixed with 10 volumes of RNAprotect, and pelleted to recover cells at room temperature. Cell pellets were resuspended in Trizol, flash frozen, and stored at -80 ^{o}C.

Additionally, due to the high frequency of Spo- mutations that occur in the *spo0A* gene in *B. anthracis* cultures [2, 3], we performed growth curves and RNA extractions of an Ames35-Spo0A mutant (Spo- strain) in parallel, and two replicates of libraries prepared from this strain in parallel were used for comparison, as described below.

Methodology: RNA samples were prepared from Trizol pellets by glass bead homogenization and use of the All Prep DNA/RNA kit (Qiagen) as described in detail in McKenzie et al. [4]. Ribosomal RNA was depleted with the MicrobEnrich kit (Life Technologies) and libraries were prepared using the TruSeq DNA Sample Preparation kit (Illumina) as described in detail in McKenzie et al. [4]. Libraries were run as 2x100 bp paired-end on two lanes of an Illumina Hiseq 2500 sequencer, generating on average 14.25 million reads per sample. Raw reads were trimmed for adapter sequence, poor quality sequence, and filtered for overall low quality using the Fastx Toolkit suite of tools (Hannon Lab, CSHL). Remaining reads were mapped to the *Bacillus anthracis* str. 'Ames Ancestor' genome (NC_007530.2) and plasmid pX01 (NC_007332.2) using Bowtie2 [5], using default parameters except using --no-mixed. Reads mapping to genes were counted using a proprietary perl script. Differential gene expression between two wild-type and two ΔHfq3 replicates or two Spo0A mutant replicates was determined using the exactTest function in EdgeR [6].

Presentation of Data Table: In the data table below, we selected the genes that were scored as differentially expressed by the EdgeR analysis method. The log_2(Fold change) is presented for the comparison of ΔHfq3 to wild-type, so positive numbers indicate increased gene expression, or upregulation as compared to wild-type Ames 35, and negative numbers indicate decreased gene expression in the comparisons, or downregulation. We also looked to see if each gene was scored as showing a significant difference in expression in the Spo0A mutant vs. wild-type Ames35 comparison. Genes with a "no" in the Spo0A column were only significantly different in the Hfq3, but not the Spo0A, mutants. While the Hfq3 mutant strains that were analyzed here were Spo+, this analysis selects for gene candidates that are the least likely to have differential expression due to the occurrence of spontaneous Spo- mutants in the cell population.

We highlighted genes that were significantly differentially expressed (as compared to wild-type) only in the Hfq3 mutant strain in light blue; medium blue highlighting indicates that the log_2(Fold change) for the Hfq3 mutant comparison and the log_2(Fold change) for the Spo0A mutant comparison were at least 2 units apart. These medium blue genes, then, represent the preliminary candidates of greatest interest for future studies.

The expression analysis also confirmed that Hfq3 was expressed in the cells and that no Hfq3 transcripts were observed in the Hfq3 mutant.

Analysis: Multiple metabolic genes (especially fatty acid metabolism genes) and membrane-associated and/or transporter genes were identified from this preliminary analysis as potentially being differentially expressed. We show here only the subset of differentially expressed genes that were upregulated greater than 5.6-fold (2^2.5) or downregulated greater than 5.6-fold in the Hfq3 mutant. Many more candidates of interest were identified as being upregulated (vs. downregulated) in the Hfq3 mutant, which would suggest that Hfq3 has a negative effect on gene expression of these genes, consistent with a model by which Hfq binds to RNAs in the cell and accelerates their degradation. Alternatively, this could be explained by regulation of the synthesis of a transcriptional regulator. 26 genes are highlighted as medium blue, of particular interest as candidates; this includes 13 uncharacterized protein-encoding genes.

Additionally, we included the decrease in gene expression observed for the MreBCD proteins, which was below the 5.6-fold threshold, but whose expression was statistically significant for each of the genes of this operon, and distinct from the regulatory profile in the Spo- comparison strain. For example, the expression level of the essential gene *mreC* was 3.7-fold higher in the wild-type Ames 35 strain as compared to the Hfq3 mutant.

We also conducted a SNP analysis on the reads from the RNA-Seq experiment, providing a look at the variability of the mutant strains. With a mutation rate in the range of 0.001-0.004 base changes per generation, >1 SNP would be expected during the creation and passage of the mutant strains. The second table below depicts non-synonymous SNPs that were identified in the mutant strains as compared to the Ames Ancestor genome that was used for alignment. Two SNPs were noted in the wild-type strain that are also observed in each of the other mutants, confirming that the mutants were derived from this parent. Note that in the Ames35-ΔHfq3 strains, two additional SNPs were noted, one in a hypothetical protein and one in a glutaminase gene. Future analysis might be best done by moving the Hfq3 deletion to another background or by whole-genome sequencing of a series of mutant isolates to identify cultures that are 100% isogenic to the wild-type for comparison, to confirm that these differentially expressed gene candidates are indeed the result of the Hfq3 deletion.

Differentially Expressed Gene Candidates: Comparison of wild-type Ames 35 and Ames 35-ΔHfq3

Original Gene Tag	Locus Tag (New Nomenclature)	Gene Description	Log_2(Fold Change)	Sig. Change in ΔSpo0A?
GBAA_1061	GBAA_RS05565	Lipoprotein	4.7	Yes, 4.8
GBAA_4175	---	Uncharacterized protein	4.4	No
GBAA_0660	GBAA_RS03585	Oligopeptide ABC transporter, ATP-binding protein	4.3	No
GBAA_0659	GBAA_RS03580	Oligopeptide ABC transporter, ATP-binding protein	4.3	No
GBAA_2552	GBAA_RS12615	**yngE**; Carboxyl transferase domain protein	4.1	No
GBAA_4176	GBAA_RS20285	Hypothetical protein	4.0	Yes

GBAA_4240	GBAA_RS20590	**thlA**; Acetyl-CoA acetyltransferase	4.0	No
GBAA_2551	GBAA_RS12610	**yngF**; Enoyl-CoA hydratase	4.0	No
GBAA_3094	GBAA_RS15180	Uncharacterized membrane protein	4.0	Yes, 2.7
GBAA_0658	GBAA_RS03575	**oppB**; Oligopeptide ABC transporter, permease protein	3.9	No
GBAA_0556	GBAA_RS03080	**yuaF**; Phosphate ABC transporter permease	3.9	No
GBAA_2547	GBAA_RS12590	Acyl-CoA dehydrogenase	3.8	No
GBAA_1091	GBAA_RS05705	Long-chain fatty acid CoA ligase	3.7	No
GBAA_2550	GBAA_RS12605	**mvaB**; Hydroxymethylglutaryl-CoA ligase	3.7	No
GBAA_0557	GBAA_RS03085	**yuaG**; Flotillin family	3.6	No
GBAA_3093	GBAA_RS15175	Membrane permease	3.6	No
GBAA_5590	GBAA_RS27230	**fadF**; Ferredoxin, 4Fe-4S	3.6	Yes, 3.3
GBAA_3095	GBAA_RS15185	LamB/YcsF family protein	3.6	Yes, 2.8
GBAA_0657	GBAA_RS03570	Oligopeptide ABC transporter, permease protein	3.5	No
GBAA_2548	GBAA_RS12595	**accC2**; Acetyl-CoA carboxylase biotin carboxylase subunit	3.5	No
GBAA_1329	GBAA_RS06775	**phaR**; Polyhydroxyalkanoid acid synthase subunit	3.5	No
GBAA_3096	GBAA_RS15190	**kipA**; Urea amidolyase	3.4	Yes, 3.0
GBAA_5249	GBAA_RS25620	**fadN**; 3-hydroxyacyl-CoA dehydrogenase	3.4	Yes, 4.1
GBAA_2296	GBAA_RS11405	CoA-transferase subunit beta	3.3	No
GBAA_4799	GBAA_RS23310	Uncharacterized protein	3.3	No
GBAA_2549	GBAA_RS12600	Acetyl-CoA carboxylase biotin carboxyl carrier protein subunit	3.3	No
GBAA_0312	GBAA_RS01760	**metN2**; Methionine import ATP-binding protein	3.2	Yes, 2.7
GBAA_5589	GBAA_RS27225	Acetyl-CoA acetyltransferase	3.2	Yes, 2.8
GBAA_0656	GBAA_RS03565	**oppA**; Oligopeptide ABC transporter substrate binding protein	3.2	No
GBAA_5536	GBAA_RS26975	**nuoJ**; NADH dehydrogenase subunit J	3.1	No
GBAA_2118	GBAA_RS10560	Uncharacterized membrane protein	3.1	No
GBAA_4617	GBAA_RS22450	Uncharacterized protein	3.1	Yes, 3.3
GBAA_0540	GBAA_RS02990	Uncharacterized protein	3.1	No
GBAA_3645	GBAA_RS17720	Oligopeptide ABC transporter substrate-binding protein	3.1	No
GBAA_5538	GBAA_RS26985	**nuoH**; NADH dehydrogenase subunit H	3.1	Yes, 2.2

GBAA_5539	GBAA_RS26990	**nuoD**; NADH dehydrogenase subunit D	3.0	Yes, 2.5
GBAA_4927	GBAA_RS23920	Hypothetical protein	3.0	No
GBAA_4800	GBAA_RS23315	Hypothetical protein	3.0	No
GBAA_0174	GBAA_RS01000	ABC transporter ATP-binding protein	3.0	Yes, 3.1
GBAA_4928	GBAA_RS23925	Uncharacterized protein	2.9	No
GBAA_4174	GBAA_RS20275	Hypothetical protein	2.9	No
GBAA_5123	GBAA_RS24880	**glgB**; Glycogen branching protein	2.9	Yes, 3.9
GBAA_0887	GBAA_RS04780	**eag**; S-layer protein EA1	2.9	No
GBAA_0604	GBAA_RS03310	**yoaR**; Vancomycin resistance protein	2.9	Yes, 2.8
GBAA_1330	GBAA_RS06780	**fabG**; Acetoacetyl-CoA reductase	2.9	No
GBAA_5542	GBAA_RS27005	**nuoA**; NADH dehydrogenase subunit A	2.9	Yes, 2.8
GBAA_5588	GBAA_RS27220	**mmgB**; 3-hydroxyacyl-CoA dehydrogenase	2.9	Yes, 2.6
GBAA_5535	GBAA_RS26970	**nuoK;** NADH dehydrogenase subunit K	2.9	No
GBAA_3646	GBAA_RS17725	Hypothetical protein	2.9	No
GBAA_2553	GBAA_RS12620	**acsA;** Acetoacetyl-CoA synthase	2.9	No
GBAA_5587	GBAA_RS27215	Acyl-CoA dehydrogenase	2.8	No
GBAA_1331	GBAA_RS06785	**phaC;** poly(R)-hydroxyalkanoic acid synthase subunit	2.8	No
GBAA_5248	GBAA_RS25615	**fadA**; Acetyl-CoA acetyltransferase	2.8	Yes, 3.3
GBAA_5523	GBAA_RS26905	Hypothetical protein	2.8	No
GBAA_3488	GBAA_RS16995	Hypothetical protein	2.8	No
GBAA_2029	GBAA_RS10125	Uncharacterized secreted protein	2.8	No
GBAA_3110	GBAA_RS15250	Histidine transporter	2.8	No
GBAA_5537	GBAA_RS26980	**nuoI;** NADH dehydrogenase subunit I	2.8	No
GBAA_3491	GBAA_RS17010	Uncharacterized membrane protein	2.8	No
GBAA_0313	GBAA_RS01765	ABC transporter permease	2.7	Yes, 2.6
GBAA_4204	GBAA_RS20415	Short chain dehydrogenase	2.7	Yes, 2.7
GBAA_0894	GBAA_RS04810	Enoyl-CoA hydratase	2.7	No
GBAA_3258	GBAA_RS15915	**yvqJ**; MFS transporter	2.7	Yes, 4.5
GBAA_5540	GBAA_RS26995	NADH dehydrogenase subunit C	2.7	Yes, 2.7
GBAA_4060	GBAA_RS19730	Uncharacterized N-acetyltransferase	2.7	Yes, 2.9
GBAA_0186	GBAA_RS01055	Oligopeptide ABC transporter permease	2.7	Yes, 2.2
GBAA_4514	GBAA_RS21960	**cccA**; Cytochrome c-550	2.6	Yes, 3.0
GBAA_5046	GBAA_RS24500	Hypothetical protein	2.6	No
GBAA_3712	GBAA_RS18050	**hutH**; Histidine ammonia-lyase	2.6	Yes, 2.7

GBAA_5496	GBAA_RS26775	ABC transporter ATP-binding protein	2.6	No
GBAA_5497	GBAA_RS26780	**yknX**; RND family efflux transporter MFP subunit	2.6	No
GBAA_4893	GBAA_RS23760	**ppnK**; Inorganic polyphosphate/ATP-NAD kinase	2.6	No
GBAA_5656	GBAA_RS27545	**mdeA**; Methionine gamma-lyase	2.6	Yes, 2.4
GBAA_0175	GBAA_RS01005	**metQ**; ABC transporter substrate-binding protein	2.6	Yes, 2.8
GBAA_3043	GBAA_RS14940	Uncharacterized protein	2.6	No
GBAA_5494	GBAA_RS26765	**yknW**; Uncharacterized membrane protein	2.6	No
GBAA_3713	GBAA_RS18055	**hutP**; histidine utilization antiterminator	2.6	Yes, 2.7
GBAA_4915	GBAA_RS23860	**acsA**; Acetyl-CoA synthetase	2.5	No
GBAA_5495	GBAA_RS26770	ABC transporter permease	2.5	No
GBAA_5541	GBAA_RS27000	**nuoB**; NADH dehydrogenase subunit B	2.5	Yes, 2.7
GBAA_3046	GBAA_RS14950	Uncharacterized membrane protein	2.5	No
GBAA_5253	GBAA_RS25635	Proline dehydrogenase	2.5	No
GBAA_0643	GBAA_RS03500	**rocE;** Amino acid ABC transporter permease	2.5	Yes, 2.7
GBAA_1251	GBAA_RS06425	**trpC;** Indole-3-glycerol phosphate synthase	2.5	Yes, 3.0
GBAA_2294	GBAA_RS11395	Aminotransferase	2.5	No
GBAA_4760	GBAA_RS23130	**etfB**; Electron transfer flavoprotein subunit beta	2.5	No
GBAA_5534	GBAA_RS26965	**nuoL;** NADH dehydrogenase subunit L	2.5	No
GBAA_5045	GBAA_RS24495	Uncharacterized membrane protein	2.5	No
GBAA_1193	GBAA_RS06150	Oligopeptide ABC transporter permease	2.5	No
GBAA_5157	GBAA_RS25165	Hypothetical protein	2.5	Yes, 2.4
GBAA_2744	GBAA_RS13535	Acetyltransferase	2.5	No
GBAA_0240	GBAA_RS01300	**hppD**; 4-hydroxyphenylpyruvate dioxygenase	2.5	No
GBAA_1528	GBAA_RS07755	Uncharacterized protein	2.5	No
GBAA_2242	GBAA_RS11145	**tatA;** Twin-arginine translocation protein TatA	2.5	Yes, 2.3
GBAA_4326	GBAA_RS21020	**rbsR;** LacI family transcriptional regulator	2.5	No
GBAA_4162	GBAA_RS20215	Uncharacterized protein	2.5	No
GBAA_4865	GBAA_RS23625	Uncharacterized membrane protein	2.5	Yes, 3.0

GBAA_5655	GBAA_RS27540	**metA**; Homoserine O-succinyltransferase	2.4	Yes, 2.5
GBAA_2658	GBAA_RS13135	Integral membrane protein	2.4	No
GBAA_4872	GBAA_RS23660	Uncharacterized membrane protein	2.4	No
GBAA_2041	GBAA_RS10185	Oligopeptide ABC transporter substrate-binding protein	2.4	No
GBAA_1192	GBAA_RS06145	Oligopeptide ABC transporter permease	2.4	No
GBAA_2479	GBAA_RS12280	**ydhM**; TetR family transcriptional regulator	2.4	No
GBAA_1153	GBAA_RS05975	Uncharacterized membrane protein	2.4	No
GBAA_0845	GBAA_RS04600	**yvsH**; Amino acid permease	-3.6	No
GBAA_4909	GBAA_RS23830	Hypothetical protein	-3.3	Yes, -4.2
GBAA_4610	GBAA_RS22415	U32 family peptidase	-3.2	No
GBAA_0661	GBAA_RS03590	**glpT**; Glycerol-3-phosphate transporter	-3.0	No
GBAA_4611	GBAA_RS22420	**yrrM**; O-methyltransferase	-3.0	Yes, -2.6
GBAA_0015	GBAA_RS00115	Deoxynucleoside kinase	-3.0	No
GBAA_0785	GBAA_RS04315	Na/Pi-cotransporter family protein	-2.9	Yes, -2.4
GBAA_4430	GBAA_RS21535	Uncharacterized protein	-2.9	Yes, -2.8
GBAA_1298	GBAA_RS06635	**potB**; Spermidine/putrescine ABC transporter permease	-2.9	No
GBAA_0200	GBAA_RS01125	Carbohydrate transporter	-2.9	No
GBAA_1625	GBAA_RS08215	**queT**; Queuosine transporter	-2.8	No
GBAA_5267	GBAA_RS25705	Hypothetical protein	-2.8	No
GBAA_1403	GBAA_RS07125	**uppP2**; UDP pyrophosphate phosphatase	-2.8	No
GBAA_0683	GBAA_RS03700	**uppP3**; UDP pyrophosphate phosphatase	-2.8	No
GBAA_4501	GBAA_RS21895	Hypothetical protein	-2.7	No
GBAA_1299	GBAA_RS06640	**potC**; Spermidine/putrescine ABC transporter permease	-2.6	No
GBAA_0014	GBAA_RS00110	**dck**; Deoxynucleoside kinase	-2.6	No
GBAA_0673	GBAA_RS03650	Hypothetical protein	-2.5	No
GBAA_3896	GBAA_RS18915	Uncharacterized protein	-2.5	Yes, -2.7
GBAA_2336	GBAA_RS11600	**prsA**; Peptidyl-prolyl isomerase	-2.5	No
GBAA_3827	GBAA_RS18585	Hypothetical protein	-2.5	No
GBAA_0690	GBAA_RS03735	**brnQ1**; Branched chain amino acid ABC transporter carrier protein	-2.5	No
GBAA_1301	GBAA_RS06650	PAP2 family protein	-2.5	No
GBAA_1713	GBAA_RS08630	**fliQ**; Flagellar biosynthesis protein	-2.5	No

GBAA_4612	GBAA_RS22425	Uncharacterized protein	-2.5	No
GBAA_4684	GBAA_RS22775	**mreB;** Rod shape determining protein	-1.8	No, -1.0
GBAA_4683	GBAA_RS22770	**mreC**; Cell shape determining protein	-1.9	No, -0.8
GBAA_4682	GBAA_RS22765	**mreD;** Rod shape determining protein	-1.9	No, -0.6

Non-synonymous SNPs Present in Strains

Genome Location	SNP Type	WT Strain	Hfq3 Mutant	Spo0A Mutant
GBAA_0995	Nonsynonymous Coding	T	T	T
GBAA_2880	Frameshift	G	G	G
glsA-2	Nonsynonymous coding		A	
GBAA_1548	Insertion of a codon		ATAT	

<u>References for Additional Experiment 1</u>

[1]. Vrentas C, Ghirlando R, Keefer A, Hu Z, Tomczak A, Gittis AG, et al. Hfqs in *Bacillus anthracis*: Role of protein sequence variation in the structure and function of proteins in the Hfq family. Protein Sci. 2015;24:1808-19.

[2]. Sastalla I, Rosovitz MJ, Leppla SH. Accidental selection and intentional restoration of sporulation-deficient *Bacillus anthracis* mutants. Appl Environ Microbiol. 2010;76:6318-6320.

[3]. Sastalla I, Leppla SH. Occurrence, recognition, and reversion of spontaneous, sporulation-deficient *Bacillus anthracis* mutants that arise during laboratory culture. Microbes Infect. 2012;14:387-391.

[4]. McKenzie AT, Pomerantsev AP, Sastalla I, Martens C, Rickfels SM, Virtaneva K, et al. Transcriptome analysis identifies *Bacillus anthracis* genes that respond to CO_2 through an AtxA-dependent mechanism.

[5]. Langmead B, Salzberg S. Fast gapped-read alignment with Bowtie 2. Nature Methods. 2012;9:357-359.

[6]. Robinson MD, McCarthy DJ, Smyth GK. edgeR: A Bioconductor package for differential expression analysis of digital gene expression data. Bioinformatics. 2010;26:139-140.
